# Continuous and prolonged breastfeeding in wild Bornean orangutans verified with fecal proteomics

**DOI:** 10.1038/s42003-026-09968-2

**Published:** 2026-05-25

**Authors:** Nur Syamimi Makbul, Tomoyuki Tajima, Tomoko Kanamori, Noko Kuze, Takumi Nishiuchi, Anna Wong, Vijay Kumar, Takumi Tsutaya

**Affiliations:** 1 Faculty of Tropical Forestry, Universiti Malaysia Sabah, Jalan UMS, Kota Kinabalu, Sabah Malaysia; 2 Center for the Study of Co*Design, Osaka University, Suita, Osaka Japan; 3 Japan Orangutan Research Center, Suginami, Tokyo Japan; 4 College of Arts and Sciences, Tamagawa University, Machida, Tokyo Japan; 5 Research Center for Integrative Evolutionary Science, The Graduate University for Advanced Studies (SOKENDAI), Shonan Village, Hayama, Kanagawa Japan; 6 Research Center for Experimental Modeling of Human Disease, Kanazawa University, Kanazawa, Ishikawa Japan; 7 Malaysian Nature Society, JKR 641, Jalan Kelantan, Bukit Persekutuan, Kuala Lumpur Malaysia; 8 Biotechnology Research Institute, Universiti Malaysia Sabah, Jalan UMS, Kota Kinabalu, Sabah Malaysia; 9 Institute for Advanced Study, Kyushu University, Fukuoka, Fukuoka Japan

**Keywords:** Biological anthropology, Molecular ecology

## Abstract

Orangutans have a slow life history with one of the longest interbirth intervals and the lowest reported infant mortality rates among primates or even mammals. Breastfeeding is a key factor in their life history because it possibly promotes offspring health and increases maternal interbirth intervals. However, quantifying milk intake is difficult, and existing estimates for their weaning age are contradictory. Here, we use fecal proteomics to predict the breastfeeding and weaning patterns in wild Bornean orangutans in Danum Valley, Sabah, Malaysia. Age changes in milk-specific proteins identified from 20 feces of five immature individuals revealed that the orangutans in Danum Valley consistently consumed milk for ≥6.5 years after birth, consistent with the behavioral evidence as having one of the longest breastfeeding periods in mammals. Milk intake was significantly correlated with higher levels of biological defense and probiotic bacterial proteins. Mothers were not pregnant with their next offspring during the breastfeeding period. These results indicate that a continuous and long breastfeeding period is a key component of the slow life history of orangutans and shows that fecal proteomics can be applied to a wide range of wild animal populations, with the potentials to uncover novel aspects of behavior and physiology.

## Abstract (web summary)

Fecal proteomics demonstrates that wild Bornean orangutans consistently consume milk for ≥6.5 years after birth, confirming that breastfeeding is a key component of the orangutan’s slow life history.

## Introduction

Breastfeeding and weaning have important roles in the early life history of mammals^1,2^. Breast milk not only provides nutrients but also biological defense agents, such as neutrophils, immunoglobulins, and bioactive substances, which strongly support the survival and health of the infant and provide lifelong health benefits^3^. Breastfeeding and weaning affect the interbirth intervals of the mother through lactational amenorrhea^4^ and energetic costs^5,6^, thereby influencing the reproductive speed and strategy^1,7^.

To investigate the significance of breastfeeding and weaning in life history, orangutans are a critical research subject^8,9^. Orangutans are the only existing great ape species that live outside of Africa. They diverged from the human ancestral clade 12–16 million years ago^10^ and consist of three existing species. Orangutans are distributed only in Borneo and Sumatra and are critically endangered, according to the IUCN Red List. The interbirth interval of wild orangutans is typically 7.6 ± 2 years^9^, which is among the longest intervals in primates^11^. Orangutan infants exhibit suckling from the mother’s nipple until 6–7 years old^12^, which represents a longer breastfeeding period than most other mammalian species, including chimpanzees (4–5 years:^13,14^), African elephants (5 years:^1^), or whales (3–4 years for killer whale^15^ and northern bottlenose whales^16^). Along with these slow life history traits, wild orangutans have low infant mortality rates compared with other apes, including most human populations^9^.

Therefore, we hypothesize that prolonged periods of milk intake, which provide enhanced biological defense agents in juveniles and contribute to the long interbirth interval for adult females, underlie the success of the slow life history of orangutans. However, the accurate assessment of quantitative or even qualitative milk intake in wild primates is difficult when using conventional behavioral proxies. Nipple suckling without the ejection of milk (i.e., “comfort nursing” or “non-nutritive suckling”) is commonly assumed to exist and possibly results in an overestimation of milk intake^17^. Non-observable breastfeeding behavior occurs in the higher canopy or at nighttime, which results in the underestimation of the period of milk intake. Although the application of isotopic or elemental proxies can reveal the actual consumption patterns of breast milk^18^, they tend to be complicated by other factors, such as dietary contents and nutritional stress, which hinder the accuracy of these proxies (see Discussion).

In this study, we examined the breastfeeding and weaning patterns of wild Bornean orangutans (*Pongo pygmaeus morio*) to understand the physiological and molecular basis for their slow life history. We used fecal proteomics as a method to more accurately reveal breastfeeding and weaning patterns. Feces contain proteins and peptides originating from the host, food, and intestinal bacteria, which can be comprehensively identified by mass spectrometry-based proteomics^19^. Breast milk contains specific proteins, of which some survive in gastric and intestinal digestion and are excreted in feces^20^, along with various proteins expressed in the host digestive tract. Fecal proteomics can identify the various proteins present in feces and provide unique insights into the behaviors and physiology of the target individuals that are not obtainable by fecal metagenomics^19^. The usefulness of fecal proteomics in addressing ecological questions has been demonstrated in a proof-of-concept study using captive macaques^19^; however, it has yet to be applied to free-ranging mammalian populations. This study aims to evaluate whether fecal proteomics is an effective tool for retrospective investigations of behavior and physiology in wild populations.

## Results

### Overall identified protein profiles

Using 27 fecal samples collected from wild Bornean orangutans in Danum Valley, Sabah, Malaysia, during 31 months from 2015 to 2018 (20 from individuals aged 2.5–6.99 years old [juveniles], 3 from 7.0 to 15.49 years old [adolescents], and 4 from ≥15.5 years old [adults]: Supplementary Table 1), 1790 protein groups were identified after excluding the common laboratory and environmental contaminants (Supplementary Data 1). The protein dataset consisted of 320 orangutan, 996 plant, 416 bacterial, and 59 other protein groups. However, the identification of plant and bacterial proteins was underrepresented because of the unavoidable constraint of the proteomic database (Supplementary Note 1). The overall number of identified protein groups did not show any specific patterns across different age groups (Supplementary Fig. 1).

### Milk intake

**Fig. 1:**
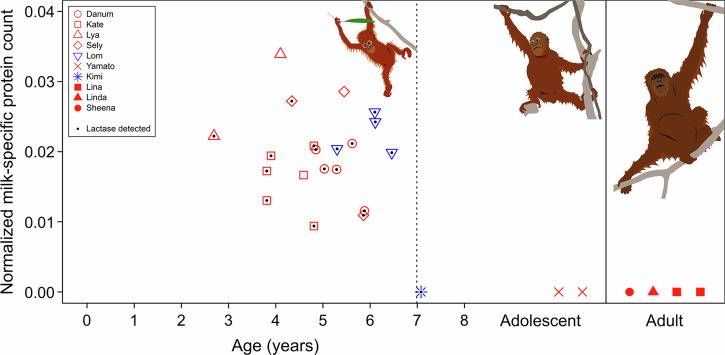
Age change in the normalized counts (i.e., divided by the number of orangutan proteins detected) of milk-specific proteins detected from the feces of orangutans in Danum Valley. Each symbol represents a different individual, with females denoted by red and males by blue. The empty and solid symbols with the same shape represent mother-offspring relationships. Samples designated as lactase are marked with a small dot at the center of the symbol. A horizontal dotted bar divides the juveniles and adolescents, and a solid bar divides the adolescents and adults. Note that the plots for >8 years on the x-axis only reflect the qualitative difference in age.

At least one milk-specific protein among alpha-S1-casein (CSN1S1), beta-casein (CSN2), kappa-casein (CSN3), and alpha-lactalbumin (LALBA) was identified from all 20 feces obtained from 5 juvenile individuals aged 2.7–6.5 years old (Fig. 1; Supplementary Fig. 2; Supplementary Table 2). Although milk contains several hundred proteins^21^, proteins specifically expressed only in the milk are those four mentioned above in the case of orangutans. These milk-specific proteins were not identified in 7 feces from 2 adolescent and 3 adult orangutan individuals (Fig. 1). Previous behavioral observations indicated that nipple-suckling behavior was observed in all infants (*n* = 7) until the age of 5 years in the same orangutan population in Danum Valley^22^. The mothers of the juveniles were not pregnant with their next sibling (i.e., a younger sister or brother) at the time of sample collection, whereas the mothers of the adolescents had already given birth to their next sibling at the time of sample collection (Supplementary Table 3).

**Fig. 2:**
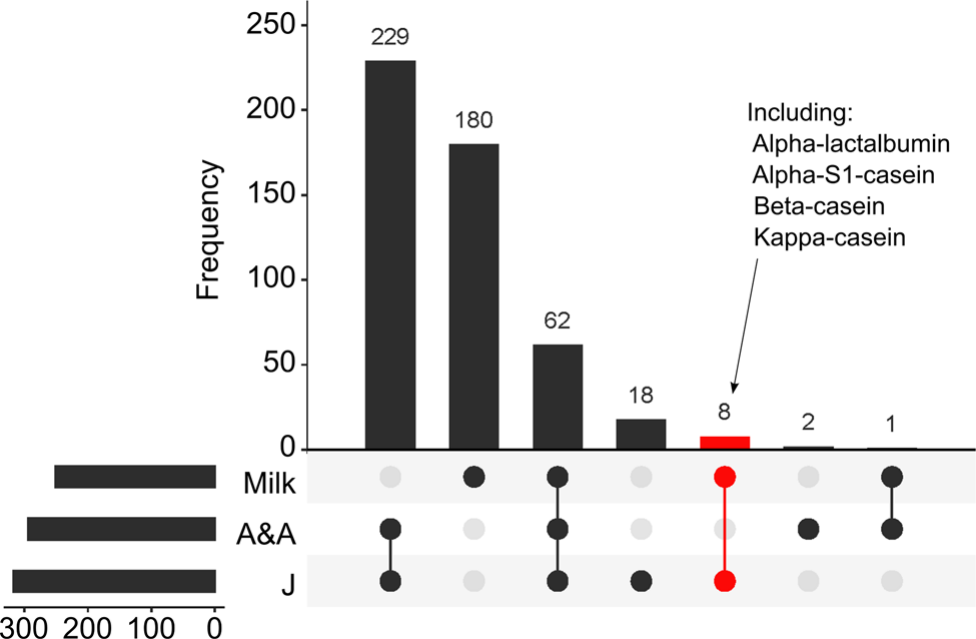
Upset plot^49^ showing the intersection of the compositions of orangutan proteins among milk (Milk), weaned adolescents and adults (A&A), and breastfed juveniles (J). The upper half of the figure presents histograms showing the number of proteins contained in each intersection of the compositions. The lower half depicts, for each histogram, the combination of compositions (represented by solid dark circles) corresponding to that intersection, indicated by connecting lines. The red bar represents the proteins identified in breast milk and juveniles but not in adolescents and adults.

Several measures support the authenticity of the identified milk-specific proteins. Previous proteomic profiling confirmed the presence of these four milk-specific proteins in milk samples from a captive Bornean orangutan individual^21^ (Fig. 2; Supplementary Data 2). Most peptide-spectrum matches of the milk-specific proteins are well-annotated (Supplementary Figs. 3–6). Because the peak retention time of the consumed food in the digestive tracts of orangutans is 50–100 h after ingestion^23^, carryover of ingested milk-specific proteins originating from a few weeks or months before sample collection is unlikely. A BLAST search showed the amino acid sequences of the identified milk-specific proteins matched those of apes or primates, rather than domesticated animals, such as cattle (Supplementary Table 4).

The expression of lactase was age-dependent and present in 90% (*n* = 18/20) of the juvenile fecal samples analyzed, but not in the adults (Fig. 1; Supplementary Table 5). Lactase is an essential enzyme to digest lactose, which is a disaccharide contained in milk, and its expression in the mammalian intestinal tract generally ceases at the end of the typical weaning age of the species^24^. Both milk-specific proteins and lactase were present in the second-oldest individual, who was 6.5 years old (Fig. 1). Lactase was detected in the feces of a 7.1-year-old individual, in the absence of milk-specific proteins (Fig. 1). The oldest sample containing lactase (7.1 years) was 7 months older compared with that of milk-specific proteins (6.5 years).

### Physiological benefits of milk

**Fig. 3:**
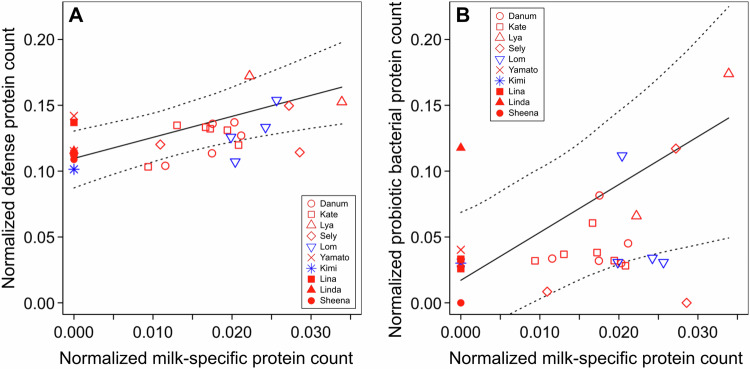
Relationship between milk-specific and other proteins. Observed and predicted relationships between the identifications of milk-specific proteins and **A** biological defense proteins or **B** probiotic bacterial proteins. Plotted symbols represent the observed results. The solid (median) and dotted (95% confidence intervals) lines represent the predicted effects estimated by LMM analyses. Each symbol represents different individuals with a red color for females and a blue color for males. The empty and solid symbols with the same shape represent mother-offspring relationships.

Analyses using linear mixed effect models (LMMs), which can account for the uneven sampling among individuals, revealed that the increase in the normalized milk-specific protein counts significantly explains the increase in biological defense protein counts (Supplementary Table 6; Supplementary Fig. 7) in the fecal samples (Fig. 3A; Supplementary Table 7) (Effect = 1.68, *p* value = 0.010). A Mann–Whitney U-test revealed that there was no significant difference in the normalized count of defense proteins in feces from juveniles and adolescents/adults (U = 95, *p* value = 0.180). The intrinsic biological defense system is not well-developed during early life, and abundant defense agents in breast milk complement this deficiency in human breastfed infants^3^. LMM analysis supported the presence of a similar physiological mechanism in wild orangutans. Given the small overall sample size and uneven sampling among individuals, these model-based inferences should be interpreted with caution, and we emphasize the qualitative patterns rather than precise effect sizes.

LMM also showed that the increase in normalized milk-specific protein counts significantly explains the increased counts of probiotic bacterial proteins from lactic acid bacteria and *Bifidobacterium* (Supplementary Table 8; Supplementary Fig. 8) in the fecal samples (Fig. 3B; Supplementary Table 9) (Effect = 3.49, *p* value = 0.025). A Mann–Whitney U-test revealed that there was no significant difference in the normalized count of probiotic bacterial proteins in feces from juveniles and adolescents/adults (U = 88, *p* value = 0.332). Lactic acid bacteria metabolize sugar to produce lactic acid to facilitate a better balance of intestinal flora and overall health^25,26^. *Bifidobacterium* dominates the intestine of healthy breastfed infants and improves their health in case of humans^27^. Breast milk contains these probiotic bacteria and promotes their establishment in the intestines of breastfed human infants^28,29^. The results of LMM analysis support the positive effects of milk on promoting the proliferation of probiotic bacteria in wild orangutans. The same limitations of small and uneven sampling described above for the LMMs apply to this result as well.

Few pathology-related bacterial proteins, such as flagellin, were identified in the feces of the subjects, and the originating microorganisms were not necessarily pathological microorganisms (Supplementary Table 10). Flagellin of *Salmonella* was identified, but species annotation was not possible because of the conserved protein sequence among various taxa (Supplementary Table 11).

## Discussion

A novel method, fecal proteomics, confirmed the continuous and prolonged breastfeeding period of orangutans, spanning over 6.5 years. This result is consistent with the previous behavioral observation and is likely more accurate given the difficulty of quantifying the suckling behaviors of highly arboreal orangutans^22^. The ≥6.5 years of breastfeeding that occurs in wild Bornean orangutans, as evidenced in Danum Valley (Fig. 1), are one of the longest among mammals^1^. The fact that the mothers of breastfed juveniles were not pregnant with their next sibling suggests that long breastfeeding periods are closely related to long birth intervals^4,7^, even in orangutans. Fecal proteomics revealed that milk intake significantly increases biological defense capacity and probiotic bacterial presence in juvenile digestive tracts (Fig. 3), which likely facilitates the health of immature orangutans. These physiological and molecular data are consistent with the ecological characteristics of orangutans, such as having one of the longest interbirth intervals and the lowest infant mortality rates^8,9^.

The results of this study also highlight the diverse life history strategies in hominids. Contrary to chimpanzees and gorillas, humans are characterized by a prolonged period of juvenile dependence and lower juvenile mortality^30^, similar to orangutans. However, humans attain these traits through cooperative childcare^31^. While accelerating weaning and shortening the birth interval for mothers, the behavioral framework of cooperative childcare, including the active provision of food to non-adults, has prevented an increase in infant/juvenile mortality in humans^32^. The behavioral basis that supports the traits of long independence and lower infant mortality is long-term, significant, energetically demanding, and a sole investment/care by the mother in orangutans^5,6^, but the active involvement of caregivers other than the mother, such as grandparents, in humans^33^.

Fecal proteomics (Fig. 1) and behavioral observations^12,22^ show the continuous and prolonged breastfeeding period in orangutans. This raises questions about the accuracy of breastfeeding and weaning patterns estimated using stable isotopic or trace elemental proxies. The nitrogen stable isotope ratio of infant body tissue increases through breast milk intake^18^. However, no stable isotopic signal of milk intake in juveniles over the age of 2.7 years was detected in wild Bornean orangutans in Danum Valley^34^. This study used the same fecal samples as those used in the previous stable isotopic study^34^, whereas milk-specific proteins were identified in feces with no increase in nitrogen stable isotope ratios (Supplementary Table 1; Supplementary Fig. 9). Because nitrogen from host cells and intestinal bacteria, as well as non-milk foods, also contributes to the nitrogen stable isotope ratios of feces, this proxy may be affected by noise^35^ and requires hundreds of matched mother-infant fecal sample pairs^13,36^ when estimating weaning patterns. Because the previous stable isotopic study did not meet this criterion^34^, the proteomic results of the present study, which provide direct evidence of milk intake, more accurately reflect the actual breastfeeding and weaning patterns of orangutans in Danum Valley. Conversely, studies using trace element concentration mapping along the growth layers of tooth enamel suggest a cyclical milk intake, with over 8 years of breastfeeding occurring in some cases, in extant museum orangutan specimens^37^ and in an extinct Pleistocene *Pongo* individual^38^. Cyclical breastfeeding was speculated to be an adaptation to the large seasonal fluctuations in food availability in the tropical rainforests of Asia and to supplement nutrition by restarting milk production when little food is available for juveniles. However, the cyclical breastfeeding pattern inferred for *Pongo* was not supported in this study, and milk-specific proteins were consistently detected in fecal samples until 6.5 years of age. Calcium-normalized barium concentrations, the inferred signal of breast milk intake^39^, are affected by nutritional stress in primates^40,41^. In Borneo, with the generally poor and fluctuating availability of energy^42,43^, nutritional stress may have masked the same trace elemental signals of breastfeeding.

Integrating these molecular and behavioral evidence provides a clearer understanding of breastfeeding and weaning patterns in wild Bornean orangutans. Fecal proteomics reveals that orangutan juveniles exhibit consistent milk intake at least from 2.7 to 6.5 years after birth (Fig. 1). However, as demonstrated by fecal stable isotope analysis, milk was not the sole source of protein during this period^34^. Although the energetic requirement to produce milk remains high in orangutan mothers^5,6^, juveniles would subsist primarily on solid foods (i.e., plants) as their main source of dietary protein during this period^34^. The seasonal availability of food plant resources fluctuates, and the nutritional status of orangutans correspondingly varies^43^, reflected in cyclic changes in the calcium-normalized barium concentrations of dental enamel^37^. Such insights could not have been obtained without fecal proteomics, which enables more sensitive detection of milk intake^19^.

The results of this study also have implications for the conservation of the critically endangered orangutan species. Recovery rates of orangutan populations are typically low due to their low population density and low reproductive rates^44^. The results of this study confirm that orangutans have prolonged periods of breastfeeding and maternal investment, which likely contribute to both high juvenile survival and low adult female reproductive rates. Shortened breastfeeding periods, caused by anthropogenic interventions or environmental changes, are likely to have long-term negative effects by either completely or partially undermining the short-term and lifelong health benefits of milk intake^3^.

## Methods

### Summary

Twenty-seven fecal samples obtained from 10 Bornean orangutan individuals (Supplementary Table 12) in Danum Valley during the 31 months from September 2015 to March 2018 were analyzed. Proteins were extracted from approximately 10 mg of the fecal samples and digested with trypsin following the protocol developed in a previous fecal proteomic study^19^. The digested peptides were purified using solid phase C18 stagetips^45^ and analyzed by liquid chromatography-tandem mass spectrometry. The resulting data were first searched against a large database (*Pongo* and Swiss-Prot excluding vertebrates) with pFind software^46^ and then searched against a cropped database, consisting of the hits from the first search, using MaxQuant software^47^. The protein groups identified with ≥2 razor+unique peptides in the second search were used for the identified protein list.

Data were analyzed in the R software environment^48^. The upset plots were drawn using the UpSetR package^49^. Generalized linear mixed models were applied using the lme4^50^ and merTools packages. To avoid bias originating from protein extraction and ionization efficiency (Supplementary Fig. 10), normalized counts, which are the number of proteins detected in a specific protein set divided by the number of proteins detected in a more general category, were used to compare samples (Supplementary Note 2).

### Ethics statements

We have complied with all relevant ethical regulations for animal use. This research was approved by the Sabah Biodiversity Center (SaBC) and Danum Valley Management Committee, with the license reference numbers JKM/MBS.1000-2/2 JLD.4 (87), JKM/MBS.1000-2/2 JLD.5 (150), and JKM/MBS.1000-2/2 JLD.15 (16). The sample transfer was approved by SaBC and the Sabah Wildlife Department with the license reference numbers JKM/MBS.1000-2/3 JLD.3 (45) and JKM/MBS.1000-2/3 JLD.5 (18). All procedures of the research followed the guideline “Code of Best Practices for Field Primatology”^51^ and were approved by the Ethics Committee for Animal Research of the Graduate University for Advanced Studies (SKD2024AR006).

### Sample and data collection

Orangutan fecal samples were collected during the 31 months from September 2015 to March 2018 in the area around Borneo Rainforest Lodge (BRL), Danum Valley Conservation Area (DVCA), Sabah, Malaysia. The study area of BRL (5°01'17“N, 117°44'50“E) is a primary forest composed predominantly of lowland dipterocarp forest. Ecological studies of wild Bornean orangutans have been carried out since 2005, and all orangutan individuals have been identified and named^52^. Fecal samples were noninvasively collected from the ground after defecation and stored inside a plastic bag during the routine behavioral observation of the subject individuals. Feces were frozen within 24 h and exported to Japan in frozen condition. Then, fecal samples were freeze-dried and stored at −30 °C until the analysis.

Life history information was obtained through the long-term study efforts in Danum Valley. Age classes of orangutans are defined as infant (0–2.49 years old), juvenile (2.5–6.99 years old), adolescent (7.0–15.49 years old), and adult (individuals aged ≥15.5 years old), based on Kuze et al.^53^. The age classes were assigned arbitrarily based on calculated age and do not necessarily reflect actual developmental stages or physiological changes in each individual. Orangutans’ ages were calculated based on the date of first appearance of the subject individual. Although the first appearance date may be later than the actual birth date, all juvenile and one adolescent individuals were estimated as neonates at the time of first appearance by assessing their physical characteristics^53^. Therefore, the unavoidable effect of underestimation of the ages was minimal in this study. The pregnancy of orangutan mothers was assessed by genital swelling^54–56^ or by subtracting the average gestation period of 7–9 months from the birth of the next offspring^54,55^.

### Protein extraction and measurement

Fecal samples were processed based on the previously reported method^19^. All experimental procedures were carried out with experimental blank samples. Approximately 10 mg of the dried feces (9.1–13.8 mg) were placed in a protein LoBind tube and suspended with 1000 μL of Gu buffer (6 M guanidium chloride, 10 mM Tris(2-carboxyethyl)phosphine hydrochloride [TCEP], 20 mM 2-Chloroacetamide [CAA], and 100 mM Tris). The samples were incubated at 90°C for 10 min, disrupted by vortexing or micropestle, and incubated again at 90°C for 5 min. The samples were centrifuged to pellet insoluble materials (2500 *×g*, 5 mins), and 700 μL of the supernatant was collected into a new protein LoBind tube. The supernatant was further centrifuged (17,000 × *g*, 2 h) to pellet bacterial fractions. The supernatant was purified by solid phase extraction using a Sep-Pak C8 cartridge. The C8 cartridge was subsequently washed with 500 μL methanol, with 80% acetonitrile (ACN) in 0.5% acetic acid and with 0.5% acetic acid. Then, 500 μL of the samples were passed through the C8 cartridges, and the cartridges were washed with 0.5% acetic acid. The samples were eluted from the C8 cartridges with 500 μL of 40% ACN in 0.5% acetic acid and then 500 μL of 60% ACN in 0.5% acetic acid. The merged eluant samples were vacuum centrifuged overnight for complete dryness. The dried fractions were resuspended with 200 μL of 50 mM triethylammonium bicarbonate (TEAB), 10 mM TCEP, and 20 mM CAA and incubated at 60 °C for 10 min. The protein concentrations were measured by BCA assay, and the solution containing 20 μg protein was taken for trypsin digestion. The subsamples were volumed up to 50 μL with 50 mM TEAB solution, mixed with 0.4 μg trypsin, and incubated at 37 °C overnight. After digestion, 10% trifluoroacetic acid (TFA) was added to bring the pH <2 to inactivate trypsin. The digested samples were desalted and purified by a stagetip with two stacked layers of solid-phase C18 membranes^45^. First, C18 stagetips were washed and equilibrated by the subsequent passing of 150 μL of methanol, 150 μL of 80% ACN in 0.1% TFA, and 150 μL of 0.1% TFA. Then, the samples were passed through the C18 stagetips, and the stagetips were washed by 0.1% TFA two times. Peptides were eluted from the C18 stagetips by passing 50 μL of 40% ACN in 0.1% TFA. The eluant samples were vacuum centrifuged for dryness and used for mass spectrometry analysis.

Liquid chromatography-tandem mass spectrometry (LC-MS/MS) analysis was performed at Kanazawa University with the condition described in Uchida-Fukuhara et al.^57^. The dried peptides were resuspended with solvent and loaded onto the nano-liquid chromatography EASY-nLC 1200 system (ThermoFisher Scientific). This system is equipped with a pre-column (Acclaim PepMap100 C18 column: inner diameter, 75 μm, length, 20 mm, particle size, 3.0 μm; ThermoFisher Scientific) and analytical column (Acclaim PepMap100 C18 column: inner diameter, 75 μm; length, 150 mm, particle size, 3.0 μm; ThermoFisher Scientific) equilibrated with 0.1% formic acid. Peptide elution is performed using a linear gradient (0–35%) of acetonitrile at a flow rate of 300 nL/min. The eluted peptides were ionized with a spray voltage of 2 kV (ion transfer tube temperature, 275 °C) and detected using LC-MS/MS (Thermo Orbitrap QE Plus, ThermoFisher Scientific) in the data-dependent acquisition mode using Xcalibur (version 4.0; Thermo Fisher Scientific). Mass spectra with 375–1500 *m/z* were obtained with a resolution of 70,000 full width at half maximum.

Proteomic data have been uploaded to the PRIDE repository^58^ with an identifier PXD060699.

### Data analysis

The .raw data files generated by LC-MS/MS were analyzed in a two-step manner. First, an open search was conducted to narrow down the protein hits for the metaproteome, using pFind software (version 3.2.0)^46^. Protein sequence data of orangutans retrieved from Uniprot (as of 2024-05-25 for *Pongo pygmaeus* and 2024-05-26 for *Pongo abelii*), entire Swiss-Prot data excluding proteins of vertebrates (as of 2025-04-25), and the common laboratory contaminants, provided by pFind, were merged and used for this first search. Swiss-Prot is a non-redundant protein amino acid sequence database with high-quality, manually annotated entries, thereby providing highly reliable protein identification results. This database encompasses a diverse range of proteins across various taxa. As of October 8, 2025, it contains 573,661 entries, including 199,540 from eukaryotes (34.8%), 336823 from bacteria (58.7%), 19,814 from archaea (3.5%), and 17,484 from viruses (3.0%). For the pFind search parameters, “Enzyme” was set to “Specific” for trypsin. The error tolerance was set to 10 ppm for the precursor and to 0.07 Da for the fragment ion, respectively. The variable modifications were oxidation (M), deamidation (NQ), and hydroxylation (P). Carbamidomethylation (C) was set as a fixed modification. Peptide FDR was set to 1%. The parameter “Peptide Mass” was set to 600–5000, and “Peptide Length” was set to 6–30. Protein FDR was set to 100% to include all potential proteins for the second step of database searching.

The identified proteins in the first search with ≥1 unique peptides were extracted from the initial database to create a cropped database. The .raw data files were searched against this cropped database and the common laboratory contaminant protein sequences, provided by MaxQuant, using MaxQuant software (version 2.6.3.0)^47^ to obtain the confident protein hits. The parameter “Enzyme” was set to “Specific” for trypsin. The variable modifications were oxidation (M), deamidation (NQ), pyro-Glu (EQ), and hydroxylation (P). These represent the post-translational modifications most frequently identified in the open pFind search (i.e., oxidation and deamidation), as well as a PTM essential for identifying collagens expected to be abundantly present in the fecal samples (hydroxylation). Carbamidomethylation (C) was set as a fixed modification. Error tolerances were kept at the default for orbitrap mass spectrometers. Peptide and protein FDRs were set to 1%. In MaxQuant, peptides are assigned to protein groups, and in our workflow, we treated entries corresponding to isoforms of the same gene as separate protein groups if their unique sequences were identified and MaxQuant listed them as separate entries. By sequentially applying two software programs that identify proteins using different algorithms, we enhanced the robustness of protein identification while also reducing the time required for database searches.

The resultant protein groups with ≥2 razor+unique peptides and MaxQuant score of >1, excluding contaminant or decoy hits, were conservatively regarded as identified from the subject fecal samples. Obtained peptide-spectrum matches (PSMs) of milk-specific proteins were visually checked for their spectral integrity. The homology of sequences for some parts of the identified peptides was checked by BLAST search.

Previously reported .raw files^21^ for breast milk samples of a captive orangutan individual were also analyzed in the same two-step manner. The cropped database for milk proteins resulting from the pFind search was used for the MaxQuant subsequent analysis.

Some proteins were classified as milk-specific, biological defense, or probiotic bacterial proteins (Supplementary Data 1). Milk contains hundreds of proteins^21^, but most of them are expressed in other biological tissues. Such proteins are not the exact proxy of milk intake. On the other hand, milk-specific proteins, which are exclusively expressed in milk, serve as a precise proxy. The four proteins, alpha-S1-casein (CSN1S1), beta-casein (CSN2), kappa-casein (CSN3), and alpha-lactalbumin (LALBA), were defied as milk-specific proteins in orangutans. Except for cancer and other disease states, biological defense proteins were classified as those involved in immunity and inflammatory response. Probiotic bacterial proteins were those from lactic acid bacteria and *Bifidobacterium*.

### Statistical tests

Since the protein extraction efficiency in the experiment processes and the ionization efficiency in the mass spectrometry differ for each sample, using the total number of identified proteins introduces bias for inter-sample comparison. For example, in the fecal samples where many orangutan protein groups were detected, the number of bacterial protein groups detected was also high (Supplementary Fig. 6). To avoid such bias, normalized protein count, which is the number of proteins detected in a specific protein set divided by the number of proteins detected in a more general category, was used when comparing samples. For example, the number of detected milk-specific proteins and biological defense proteins was normalized by dividing by the total number of detected orangutan proteins in the same sample, and the number of detected probiotic bacterial proteins was normalized by dividing by the total number of detected bacterial proteins in the same sample.

Identified protein data were analyzed using the R software environment (version 4.2.3)^48^. Package UpSetR was used to draw upset plots^49^. Packages lme4 (version 1.1-31)^50^ and merTools (version 0.6.2)^59^ were used for generalized linear mixed models. Statistical significance was set as α < 0.05. R scripts used to analyze data have been uploaded to the Zenodo repository^60^.

Linear mixed-effect models (LMMs) were constructed to investigate the effects of breast milk intake on the health status of the digestive tracts. We tested the following two hypotheses:

- Increased biological defense by the breast milk intake: The intrinsic biological defense system is not well-developed during early life, and abundant defense agents in breast milk complement this deficiency in human breastfed infants^3^. If a similar mechanism exists in orangutans, a greater intake of breast milk enriches biological defense proteins in the digestive tract, thereby feces.
- Increased probiotic bacterial presence/activities by the breast milk intake: Lactic acid bacteria metabolize sugar to produce lactic acid and facilitate a better balance of intestinal flora and overall health^61^. *Bifidobacterium* dominates the intestine of healthy breastfed infants and improves their health conditions^27^. Breast milk contains these probiotic bacteria and promotes their establishment in the intestines of breastfed human infants^28,29^. If a similar mechanism exists in orangutans, a greater intake of breast milk enriches probiotic bacterial proteins in the intestinal tract, thereby feces.

To test these hypotheses, we constructed the models considering the normalized milk-specific protein counts (continuous variable), age category (juvenile, adolescent, or adult), and sex (female or male) as fixed effects and individuals (1|name) as a random effect for the analyses of the normalized counts of biological defense proteins (Model 1) or probiotic bacterial proteins (Model 2). While LMMs are well-suited for handling clustered and unbalanced data, small and uneven sample sizes can reduce the robustness of the model assumptions and increase the uncertainty of parameter estimates. For this reason, the LMM results should be treated with appropriate caution, and the qualitative pattern, rather than precise effect sizes, should be emphasized.

### Reporting summary

Further information on research design is available in the Nature Portfolio Reporting Summary linked to this article.

## Supplementary information

Supplementary Information

Description of Additional Supplementary Materials

Supplementary data 1

Supplementary data 2

Reporting Summary

Transparent Peer Review file

## Data Availability

Proteomic data were uploaded to the PRIDE repository^58^ with an identifier PXD060699. Other data is reported in the Supplementary Information.

## Code availability

R scripts used to analyze data have been uploaded to the Zenodo repository with the digital object identifier 10.5281/zenodo.17309234^60^.
