# Supplementary material for: Continuous and prolonged breastfeeding in wild Bornean orangutans verified with fecal proteomics
Source: Commun Biol. 2026 May 25;9:973. doi: 10.1038/s42003-026-09968-2 (PMC13376732; doi:10.1038/s42003-026-09968-2)
Supplement: Supplementary file 1 — Supplementary Information [file 42003_2026_9968_MOESM1_ESM.pdf]

Supplementary Information for
Continuous and prolonged breastfeeding in wild Bornean orangutans verified with fecal proteomics

Nur Syamimi Makbul*, Tomoyuki Tajima, Tomoko Kanamori, Noko Kuze, Takumi Nishiuchi, Anna Wong, Vijay Kumar, Takumi Tsutaya*

Correspondence to:

Nur Syamimi Makbul (syamimimakbul@gmail.com)
Takumi Tsutaya (tsutayatakumi@gmail.com)

This PDF file includes:

Supplementary Notes 1 to 2
Supplementary Figures 1 to 10
Supplementary Tables 1 to 12
Captions for Supplementary Datasets 1 to 2
Supplementary References

Other supplementary materials for this manuscript include the following:

Supplementary Datasets 1 to 2

Supplementary Note 1. Underrepresentation of plant and bacterial proteins

In bottom-up proteomics, database searches are used to match mass spectra and peptide sequences. Therefore, proteins whose sequences do not exist in the database cannot be identified even if they are present in the sample. There is a great lack of information on the protein sequences of wild orangutans' food plants and their intestinal bacteria (but see ref *1*). In this study, we used Swiss-Prot as a representative of food plants and intestinal bacteria to identify proteins. If the protein sequence is conserved among the species, homologous proteins can still be identified by bottom-up database search. The relatively large numbers of plant and bacterial proteins identified show the usefulness of this approach in this study.

However, caution is needed when interpreting the results. For example, it is highly unlikely that the wild orangutans in Danum Valley consume *Arabidopsis thaliana* (model plant organism), *Zea maize* (corn), or *Oryza sativa* (rice), while they were identified (Supplementary Data 1). Identifications of these laboratory or domesticated plants were due to the consumption of wild plants that have homologous protein sequences but are not present in the database. It should be noted that the plant and bacterial data presented in this study are suboptimal results obtained under the unavoidable constraint of the proteomic database. Although the data can properly be used for comparisons between individuals such as in this study, the accuracy and precision of the identification can be greatly improved by constructing and using more appropriate databases in the future.

Supplementary Note 2. Quantitative aspects of the proteomic data

The normalized protein count was used as an indicator when comparing samples to cancel the bias in the overall protein identification efficiency (see Methods). Among the subject fecal samples, linear models showed that the numbers of identified bacterial proteins ($R^2 = 0.782$, p-value < 0.001) and plant proteins ($R^2 = 0.679$, p-value < 0.001) were significantly positively correlated with the number of identified orangutan proteins (Supplementary Figure 10).

In label-free proteomics such as this study, the identification results are not strictly quantitative. This is because the ionization efficiency of peptides differs by their sequence and the originating samples, and the acquisition conditions in the mass spectrometry are not necessarily the same. Therefore, in this study, the number of identified proteins was used as an approximate quantitative measure. The analytical uncertainty can be canceled out by normalizing the protein count. There still is uncertainty in the original protein concentration, but this uncertainty can be overcome by targeting multiple groups of proteins that work on the same biological functions, such as milk or biological defense. Depending on the dynamic range of protein concentrations, there is a gradation of proteins that can be reliably identified (medium to high concentrations), proteins whose detectability fluctuates stochastically (low concentrations), and proteins that are rarely detected (very low concentrations). However, if the proteins are from a single substance with the same composition, such as milk, the overall probability of detection (i.e., the number of proteins) can be expected to correlate with the concentration of the original substance positively. The proteins with the low concentration are expected to have greater variations in the probability of detection, but this stochastic nature is even out as the number of proteins in the same biological function increases. In this respect, single lactase is greatly affected by stochastic effects, but the number of the four milk-specific proteins and the >20 biological defense and probiotic bacterial proteins can be relatively robust quantitative indicators.

More specifically, this issue can be rephrased as: to what extent the probabilistic false negatives inherent to protein identification influence results when the number of proteins in the category of interest is small. In proteomics, proteins in a sample may not be detected (i.e., false negatives) due to, for example, poor extraction efficiency in the lab and poor ionization efficiency in the mass spectrometer. This effect occurs randomly, but its practical importance depends on the number of target proteins. If only one protein is targeted, and it happens to be a false negative, the false negative affects 1/1 of the protein, and the impact on overall results is 100%. In contrast, with a larger target number—say, 20 proteins—a single random false negative affects only 1/20 of the proteins (5%) and the overall impact is minimal. Taking lactase as an example, which is presumed to be expressed in all juveniles below a certain age, 2 of the 20 juvenile individuals did not yield lactase detections (Figure 1); the false negative rate is thus estimated at approximately 10%. For the milk-specific protein category, which includes only four proteins, a single false negative would affect 1/4 of the proteins (25%). If the 10% false negative rate for lactase is applied, the probability that all four proteins in this category are false negatives is $0.1^4 = 0.1\%$. Therefore, if a binary standard (i.e., whether milk-specific proteins are detected) were used, one could accurately estimate breastfeeding/weaning status in 999 out of 1000 samples. On the other hand, an unknown degree of variance due to random false negatives is introduced into normalized continuous variables. The width of the predicted intervals shown in Figure 3 likely reflects, in part, such effects. Regarding LMMs, the predictor was ultimately derived from counts of a small set of milk-specific proteins (on an original scale of 0–4) that were normalized to allow comparability across samples. Thus, although the normalization yields a quasi-continuous predictor suitable for linear modeling, the underlying discreteness and limited range of the original data further reinforce the need to treat the LMM results with caution. For this reason, we used the LMM as an explanatory tool that formalizes the observed association between milk-specific and biological defense/probiotic bacterial proteins, rather than to obtain precise effect sizes. Nevertheless, even taking this into account, this study found significant associations between the normalized counts of milk-specific proteins and those of biological defense proteins and probiotic bacterial proteins. This strengthens rather than weakens the study’s argument.

We used the number of identified proteins as an approximate quantitative measure, but their intensity can also be used. Thus, the natural logarithm of the intensity (log intensity) resulting from MaxQuant analysis, adding 1 if the value was zero, was calculated and investigated. When we used the normalized intensity to cancel out the uncertainty due to the difference in ionization

efficiency, the results were similar to when we used the number of identified proteins (Supplementary Figures 1, 2, 7, 8). When using the number of identified proteins, each protein has an equal weight regardless of concentration, but when using intensity, proteins with higher concentrations have greater weights. Nevertheless, the number of detected proteins was used in this study for the main result to avoid bias towards specific high-concentration proteins and to take into account the presence of various proteins.

Supplementary Figures

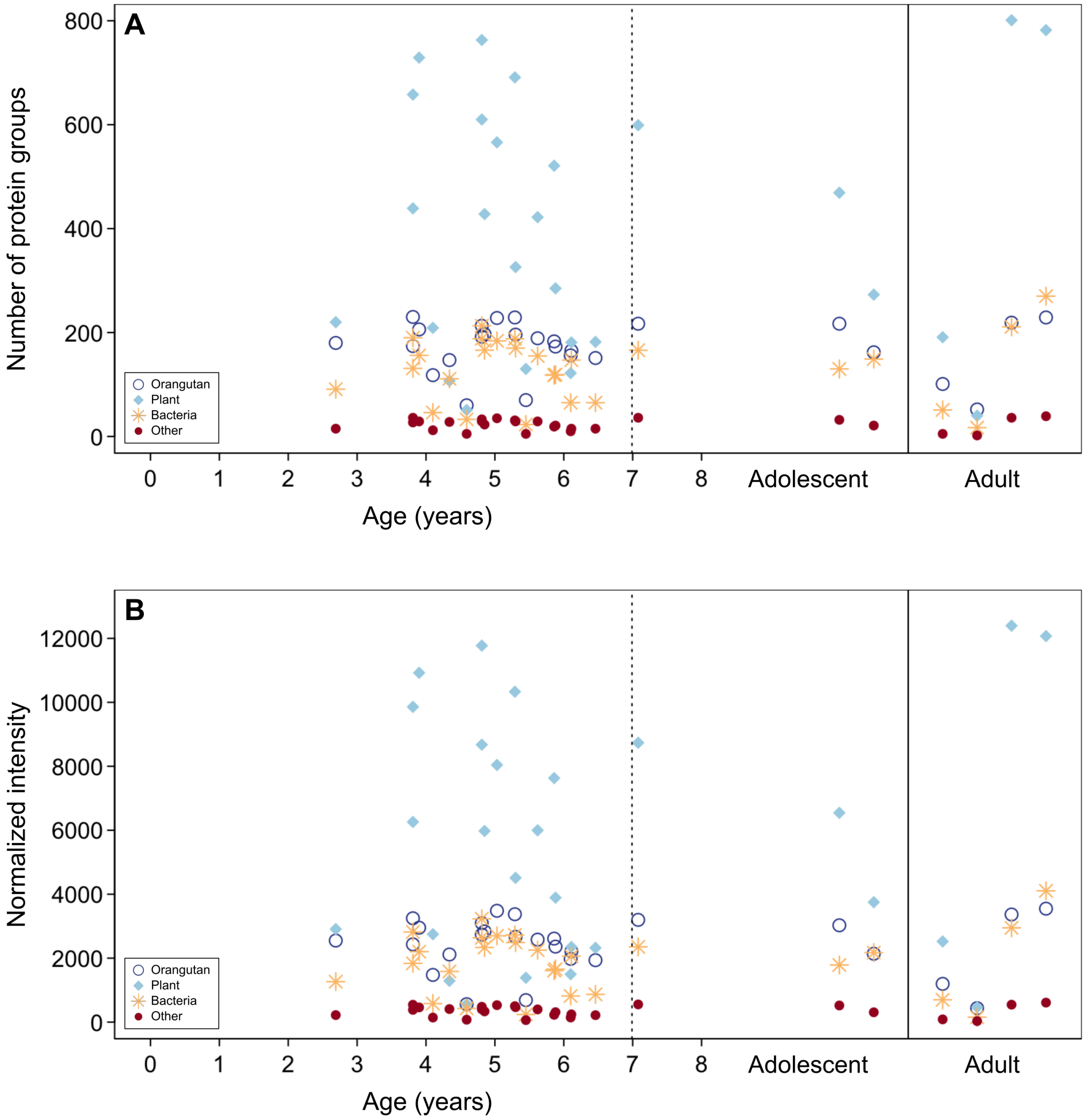

Supplementary Figure 1. Age change in **A**) the number and **B**) the normalized intensity of proteins detected from the feces of orangutans in Danum Valley. Each symbol represents different categories of proteins. A horizontal dotted bar divides juveniles and adolescents, and a solid bar divides adolescents and adults. Note that the plots for >8 years on the x-axis only reflect the qualitative difference in age.

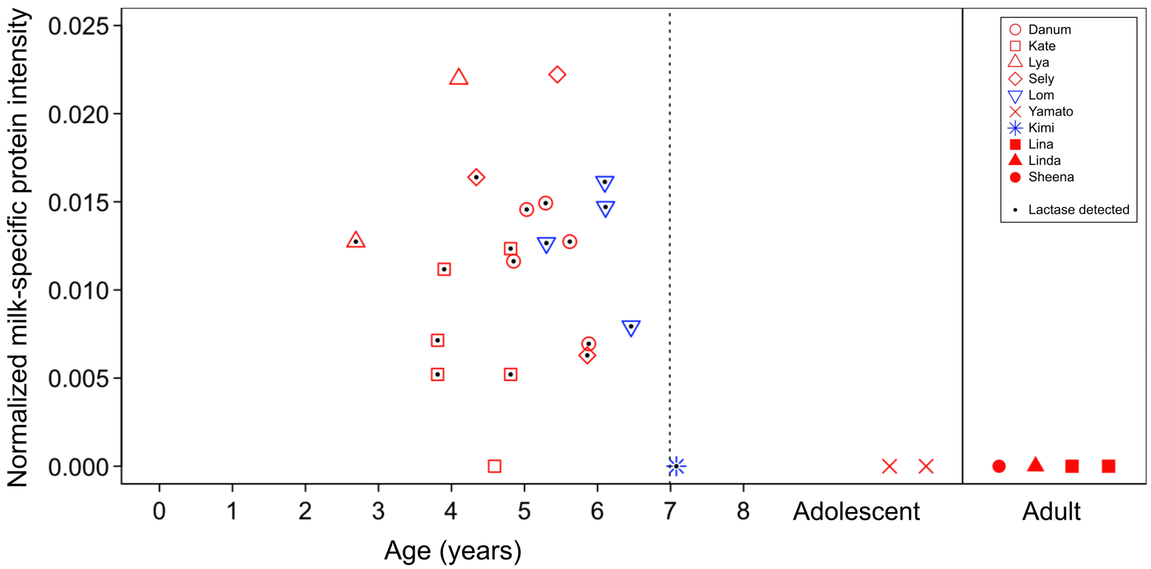

Supplementary Figure 2. Age change in the normalized intensities of the breast milk-specific proteins detected from the feces of orangutans in Danum Valley. Each symbol represents different individuals with red color for females and blue color for males. The empty and solid symbols with the same shape represent mother-offspring relationships. Samples with the identification of lactase are marked with a small dot at the center of the symbol. A horizontal dotted bar divides juveniles and adolescents, and a solid bar divides adolescents and adults. Note that the plots for >8 years on the x-axis only reflect the qualitative difference in age.

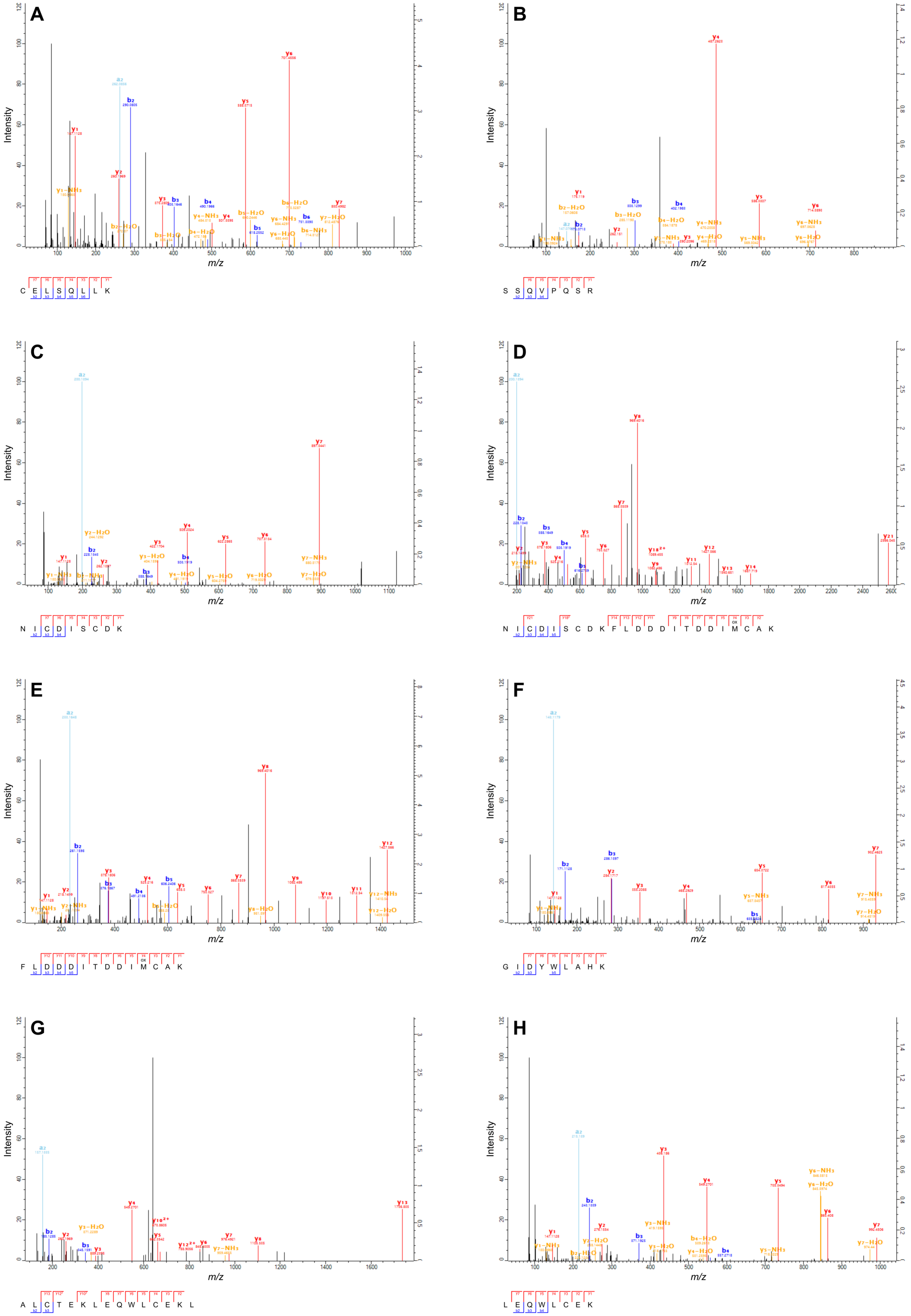

Supplementary Figure 3. Peptide-spectrum matches (PSMs) for LALBA protein identified from the feces of juvenile orangutans in Danum Valley. The upper half of each figure presents a

tandem mass spectrum, and the lower half presents the identified peptide sequence with ion annotations.

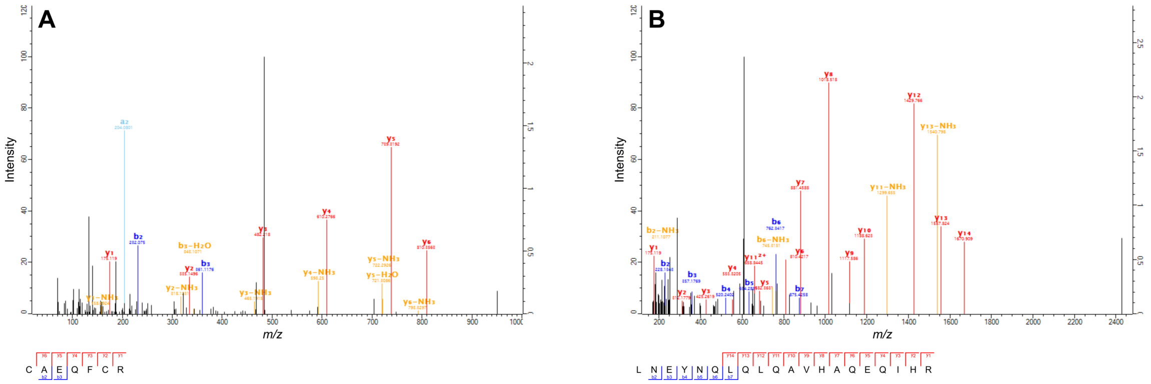

Supplementary Figure 4. PSMs for CSN1S1 protein identified from the feces of juvenile orangutans in Danum Valley. The upper half of each figure presents a tandem mass spectrum, and the lower half presents the identified peptide sequence with ion annotations.

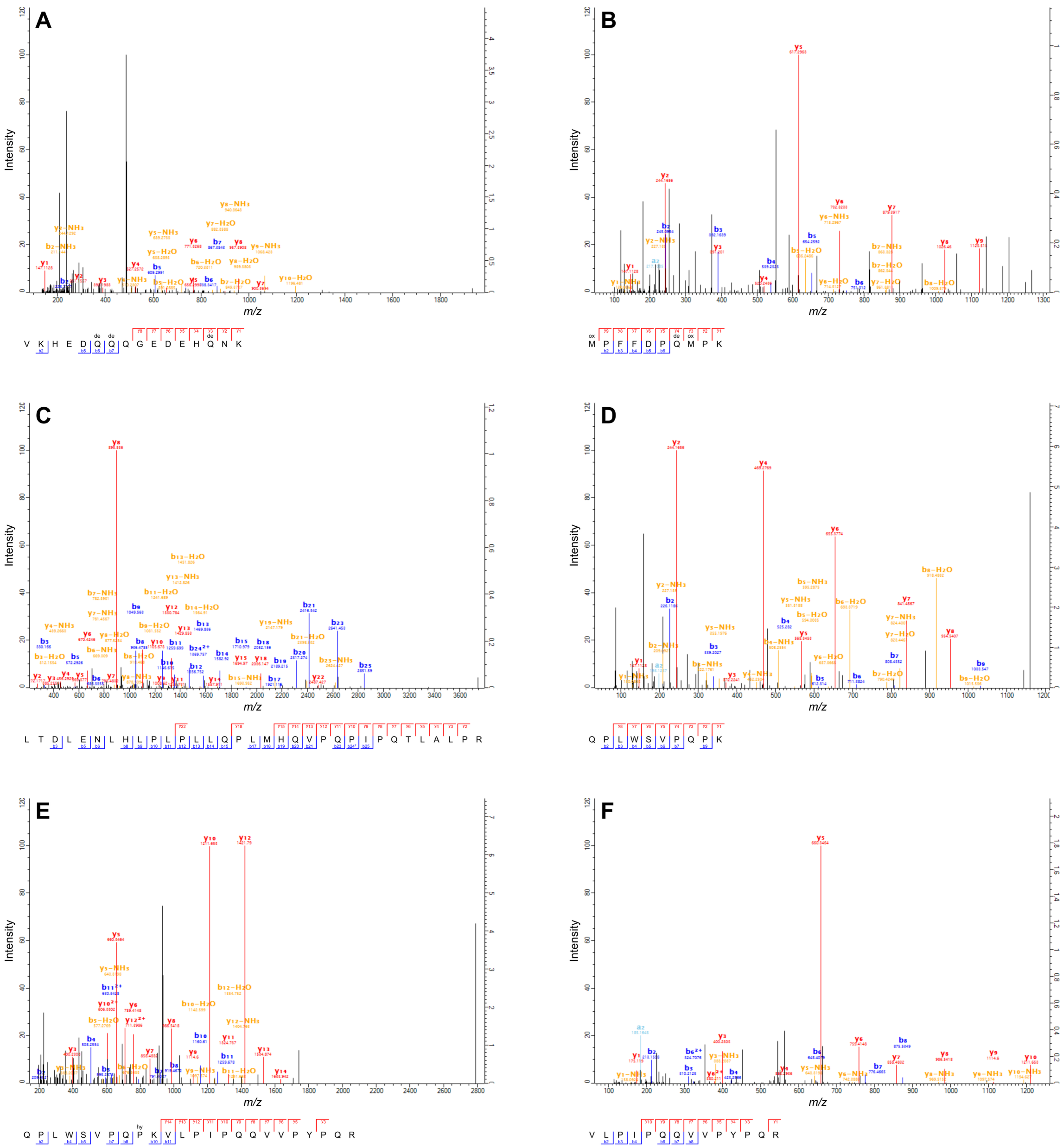

Supplementary Figure 5. PSMs for CSN2 protein identified from the feces of juvenile orangutans in Danum Valley. The upper half of each figure presents a tandem mass spectrum, and the lower half presents the identified peptide sequence with ion annotations.

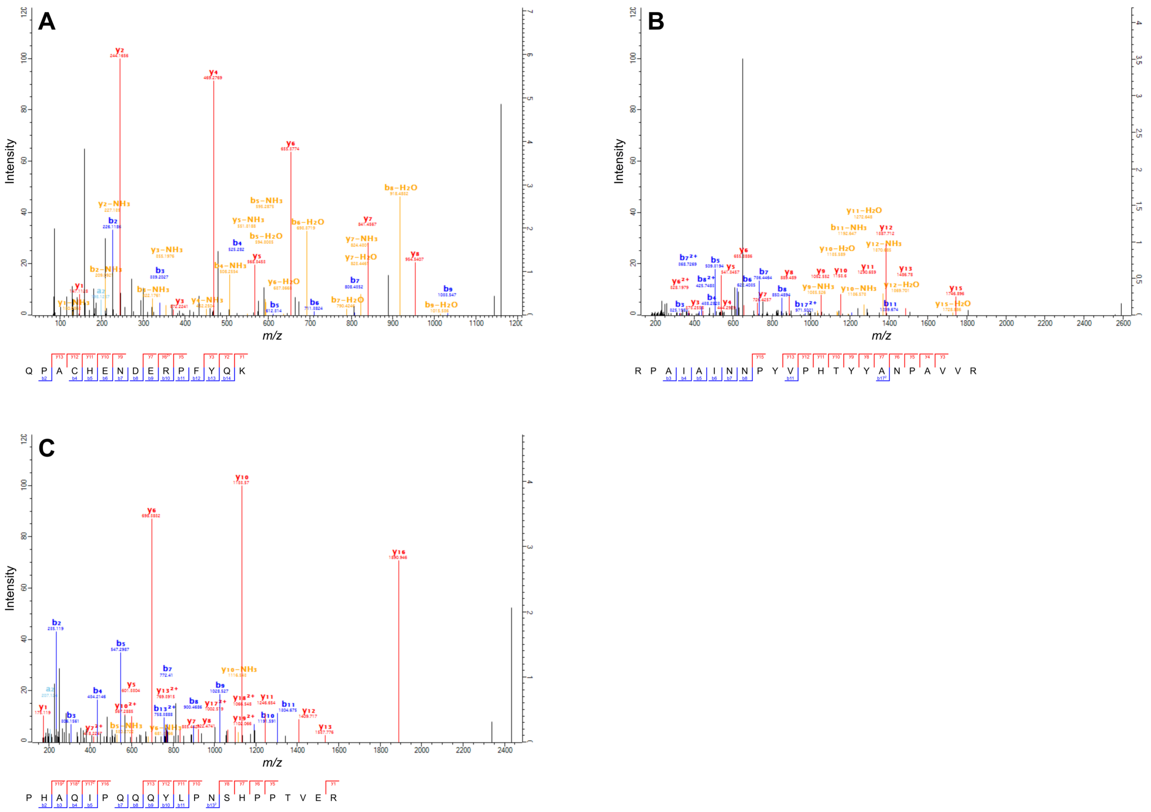

Supplementary Figure 6. PSMs for CSN3 protein identified from the feces of juvenile orangutans in Danum Valley. The upper half of each figure presents a tandem mass spectrum, and the lower half presents the identified peptide sequence with ion annotations.

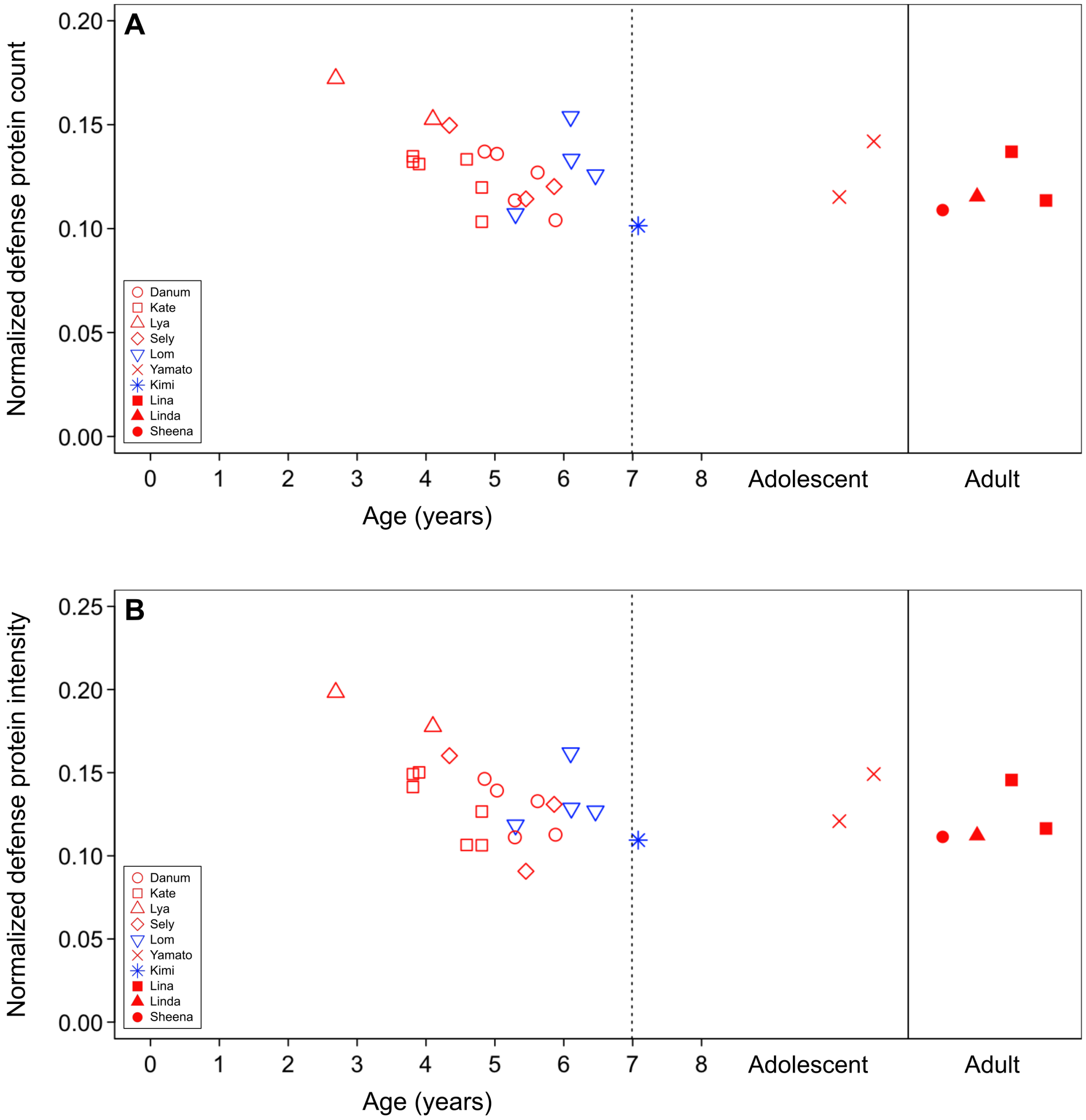

Supplementary Figure 7. Age change in **A**) the normalized counts and **B**) the normalized intensities of the biological defense proteins detected from the feces of orangutans in Danum Valley. Each symbol represents different individuals with red color for females and blue color for males. The empty and solid symbols with the same shape represent mother-offspring relationships. A horizontal dotted bar divides juveniles and adolescents, and a solid bar divides adolescents and adults. Note that the plots for >8 years on the x-axis only reflect the qualitative difference in age

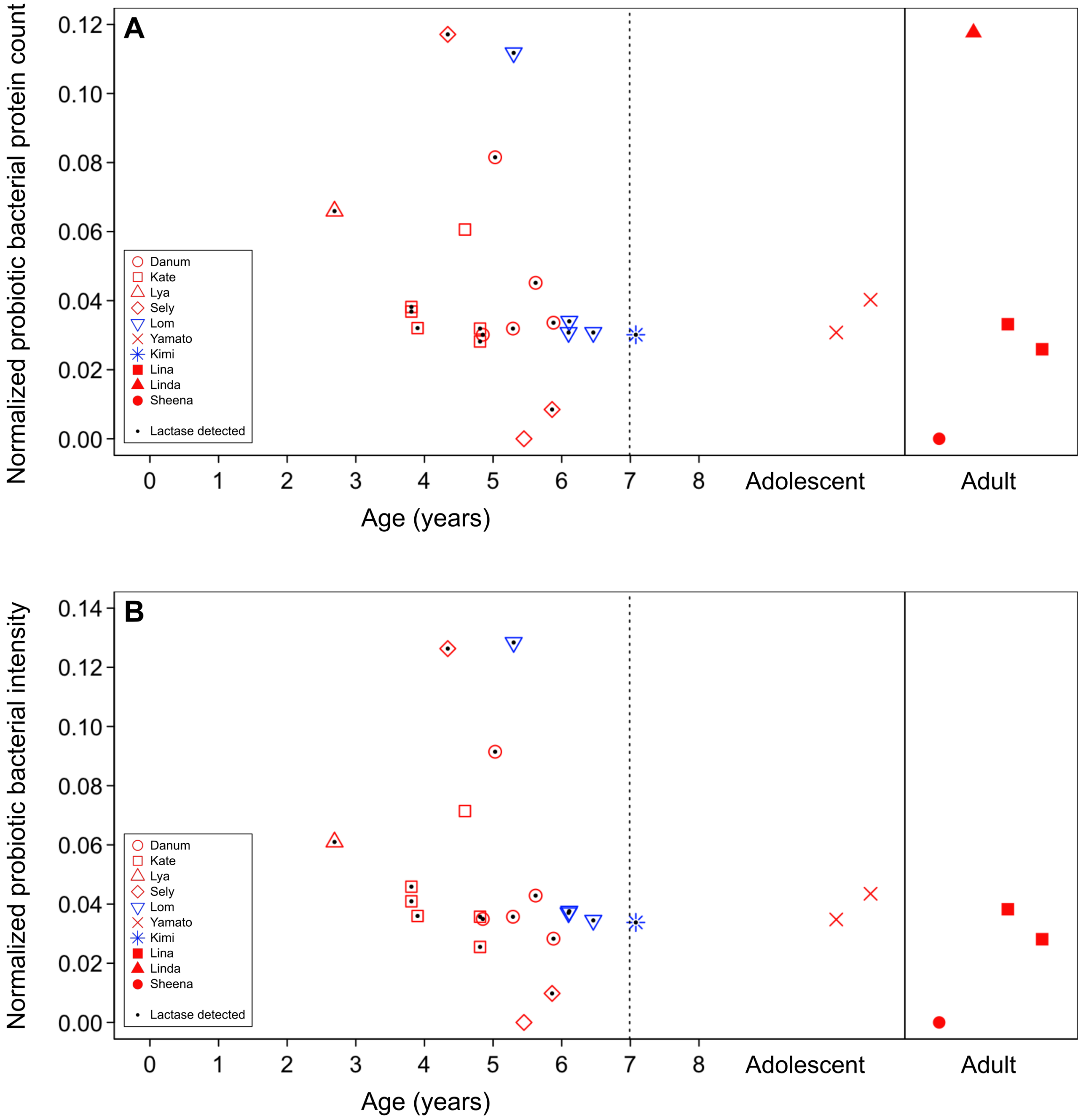

Supplementary Figure 8. Age change in **A**) the normalized counts and **B**) the normalized intensities of the probiotic bacterial proteins detected from the feces of orangutans in Danum Valley. Each symbol represents different individuals with red color for females and blue color for males. The empty and solid symbols with the same shape represent mother-offspring relationships. Samples with the identification of lactase are marked with a small dot at the center of the symbol. A horizontal dotted bar divides juveniles and adolescents, and a solid bar divides adolescents and adults. Note that the plots for >8 years on the x-axis only reflect the qualitative difference in age.

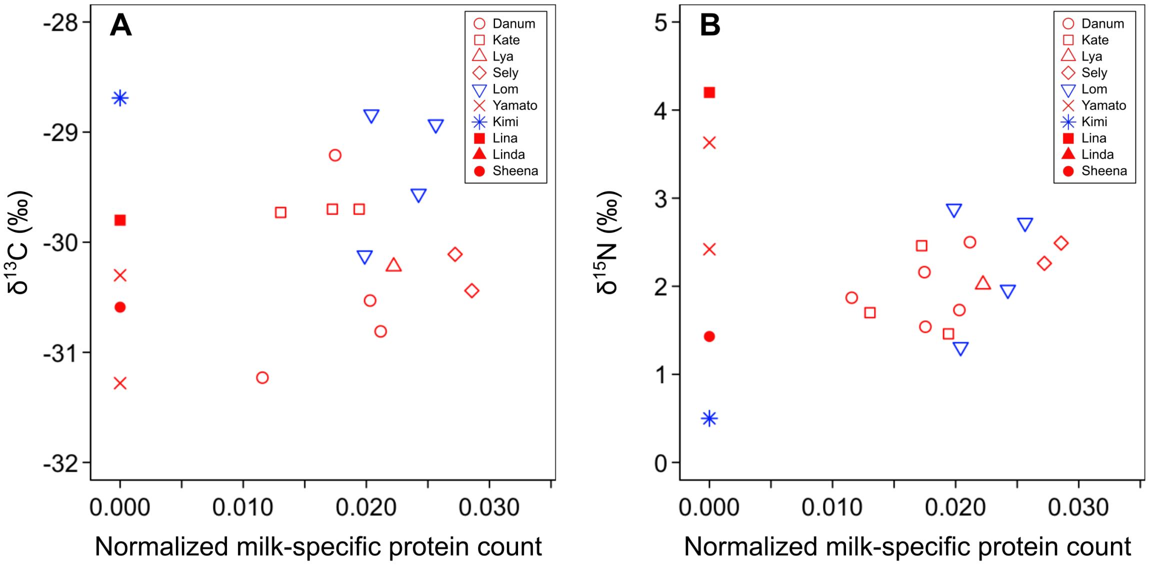

Supplementary Figure 9. Relationships between the normalized count of milk-specific proteins and **A**) the carbon or **B**) nitrogen stable isotope ratios of the feces of orangutans in Danum Valley. Each symbol represents different individuals with red color for females and blue color for males. The empty and solid symbols with the same shape represent mother-offspring relationships. Note that some fecal samples were not the subject of the previous stable isotopic study (*2*).

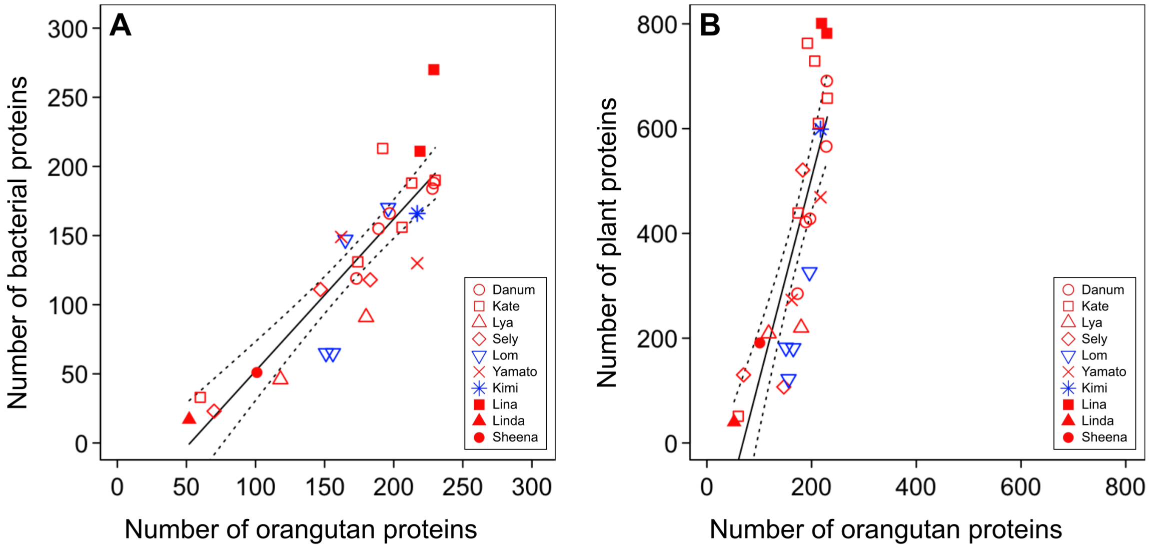

Supplementary Figure 10. Linear positive correlations between the number of identified orangutan proteins and **A**) the number of bacterial proteins or **B**) the number of plant proteins in the feces of orangutans in Danum Valley. Each symbol represents different individuals with red color for females and blue color for males. The empty and solid symbols with the same shape represent mother-offspring relationships. The solid and dotted lines show the regression line and its 95% confidence intervals, respectively. The regression lines are y = 1.0996x - 57.9374 for bacterial proteins (R^2 = 0.782) and y = 3.8603x - 265.5838 for plant proteins (R^2 = 0.679).

Supplementary Tables

Supplementary Table 1. List of the fecal samples analyzed in this study, and information on the host orangutan individuals. *: Yamato was estimated as four years old at the first appearance (2005-09-22), and her birth date was estimated from this information. Other juvenile and adolescent individuals were neonates at the first date of appearance. Fecal $\delta^{13}C$ and $\delta^{15}N$ values were referred from Tsutaya et al. (*2*).

ID	Collection date	Name	Age class	N. of detected milk-specific proteins	Sex
DD2016-62	2016-10-18	Lya	Juvenile	3	Female
DD2016-21	2016-05-06	Kate	Juvenile	3	Female
DD2016-23	2016-05-07	Kate	Juvenile	3	Female
DD2016-46	2016-06-06	Kate	Juvenile	3	Female
DD2018-13	2018-03-15	Lya	Juvenile	4	Female
DD2015-06	2015-10-06	Sely	Juvenile	4	Female
DD2017-17	2017-02-13	Kate	Juvenile	1	Female
DD2017-25	2017-05-06	Kate	Juvenile	2	Female
DD2017-27	2017-05-07	Kate	Juvenile	4	Female
DD2016-01	2016-01-07	Danum	Juvenile	3	Female
DD2016-10	2016-03-12	Danum	Juvenile	4	Female
DD2016-28	2016-06-13	Danum	Juvenile	3	Female
DD2015-04	2015-09-21	Lom	Juvenile	4	Male
DD2016-69	2016-11-16	Sely	Juvenile	2	Female
DD2016-60	2016-10-13	Danum	Juvenile	4	Female
DD2017-24	2017-04-13	Sely	Juvenile	2	Female
DD2017-05	2017-01-14	Danum	Juvenile	2	Female
DD2016-37	2016-07-12	Lom	Juvenile	4	Male
DD2016-38	2016-07-13	Lom	Juvenile	4	Male
DD2016-70	2016-11-19	Lom	Juvenile	3	Male
DD2016-15	2016-04-10	Kimi	Adolescent	0	Male
DD2016-25	2016-05-14	Yamato	Adolescent	0	Female
DD2016-39	2016-07-14	Yamato	Adolescent	0	Female
DD2016-09	2016-03-12	Sheena	Adult	0	Female
DD2016-45	2016-06-06	Lina	Adult	0	Female
DD2017-30	2017-05-12	Lina	Adult	0	Female
DD2018-12	2018-03-15	Linda	Adult	0	Female
Blank_1	–	Experimental blank	–	0	–
Blank_2	–	Experimental blank	–	0	–

Blank_3	–	Experimental blank	–	0	–
Blank_4	–	Experimental blank	–	0	–

(continued)

ID	First observation date	Age (day)	Age (year)	Amount used (mg)	$\delta^{13}C$ (‰)	$\delta^{15}N$ (‰)	.raw file
DD2016-62	2014-02-09	982	2.69	10.5	-30.22	2.02	18_DD2016_62_20240403.raw
DD2016-21	2012-07-15	1391	3.81	11.1	-29.70	2.46	7_DD2016_21.raw
DD2016-23	2012-07-15	1392	3.81	10.3	-29.73	1.70	8_DD2016_23.raw
DD2016-46	2012-07-15	1422	3.90	9.1	-29.70	1.46	16_DD2016_46_20240403.raw
DD2018-13	2014-02-09	1495	4.10	11.3	Not published	Not published	10_DD2018_13_20240403.raw
DD2015-06	2011-06-05	1584	4.34	11.8	-30.11	2.26	4_DD2015_06.raw
DD2017-17	2012-07-15	1674	4.59	9.3	Not published	Not published	5_DD2017_17_20240403.raw
DD2017-25	2012-07-15	1756	4.81	11.6	Not published	Not published	7_DD2017_25_20240403.raw
DD2017-27	2012-07-15	1757	4.81	11.3	Not published	Not published	8_DD2017_27_20240403.raw
DD2016-01	2011-03-02	1772	4.85	9.9	-30.53	1.73	5_DD2016_01.raw
DD2016-10	2011-03-02	1837	5.03	11.2	N/A	1.54	6_DD2016_10.raw
DD2016-28	2011-03-02	1930	5.29	9.6	-29.21	2.16	13_DD2016_28_20240403.raw
DD2015-04	2010-06-06	1933	5.30	10.2	-28.84	1.31	3_DD2015_04.raw
DD2016-69	2011-06-05	1991	5.45	13.8	-30.44	2.49	3_DD2016_69_20240403.raw
DD2016-60	2011-03-02	2052	5.62	10.0	-30.81	2.50	17_DD2016_60_20240403.raw
DD2017-24	2011-06-05	2139	5.86	10.8	Not published	Not published	6_DD2017_24_20240403.raw
DD2017-05	2011-03-02	2145	5.88	10.5	-31.23	1.87	19_DD2017_05_20240403.raw
DD2016-37	2010-06-06	2228	6.10	9.2	-28.93	2.72	14_DD2016_37_20240403.raw
DD2016-38	2010-06-06	2229	6.11	9.5	-29.56	1.96	15_DD2016_38_20240403.raw
DD2016-70	2010-06-06	2358	6.46	13.1	-30.12	2.88	4_DD2016_70_20240403.raw
DD2016-15	2009-03-15	2583	7.08	11.2	-28.69	0.50	2_DD2016_15_20240606.raw
DD2016-25	2005-09-22*	5347	14.65	12.3	-30.30	3.63	3_DD2016_25_20240606.raw
DD2016-39	2005-09-22*	5408	14.82	9.6	-31.28	2.42	4_DD2016_39_20240606.raw
DD2016-09	Adult	Adult	Adult	10.0	-30.59	1.43	2_DD2016_09.raw
DD2016-45	Adult	Adult	Adult	9.3	-29.80	4.20	12_DD2016_45_20240403.raw
DD2017-30	Adult	Adult	Adult	11.1	Not published	Not published	9_DD2017_30_20240403.raw
DD2018-12	Adult	Adult	Adult	12.0	Not published	Not published	2_DD2018_12_20240403.raw
Blank_1	–	–	–	0	–	–	1_Blank.raw
Blank_2	–	–	–	0	–	–	1_Blank1_20240403.raw
Blank_3	–	–	–	0	–	–	11_Balnk2_20240403.raw
Blank_4	–	–	–	0	–	–	1_Blank_20240606.raw

Supplementary Table 2. List of milk-specific proteins identified from the orangutan fecal samples.

Uniprot accession	Protein name	Organism	Gene name	Razor + unique peptides	Unique + razor sequence coverage (%)	Score
H2NH48	Alpha-lactalbumin	*Pongo abelii*	LALBA	8	44.4	115.96
H2PDG9	Alpha-S1-casein	*Pongo abelii*	CSN1S1	2	14.6	4.4654
H2PDH0	Beta-casein	*Pongo abelii*	CSN2	6	36.7	269.67
H2PDH8	Kappa-casein	*Pongo abelii*	CSN3	3	32.4	156.35

Supplementary Table 3. Observation records of the pregnancy and birth status of the mothers of the non-adult individuals targeted in this study. “1” indicates that the observation was possible in that month, and “0” indicates that it was not possible. When “P” is written with a slash, it indicates that pregnancy was observed or that it was clear that the subject individual was pregnant from the date of the next birth. The data includes the 9 months before and after the sample collection period, taking into account the gestation period of orangutans.

Mother	Lina	Sheena	Yanti	Beth	Linda	Sumi
Offspring	Kate	Danum	Sely	Lom	Lya	Yamato, Kimi
2014-12	1	0	1	0	0	0
2015-01	0	0	0	0	1	0
2015-02	0	1	0	0	1	0
2015-03	1	0	1	1	0	0
2015-04	1	0	1	1	0	0
2015-05	1	0	1	0	0	0
2015-06	1	0	1	1	0	0
2015-07	1	0	1	0	0	0
2015-08	0	0	0	0	0	0
2015-09	1	0	1	1	0	0
2015-10	1	0	1	0	0	0
2015-11	0	0	1	0	0	0
2015-12	0	1	0	0	0	0
2016-01	0	1	0	0	0	0
2016-02	0	0	1	0	0	0
2016-03	1	1	0	0	0	0
2016-04	0	1	0	0	0	1
2016-05	1	1	1	0	0	0
2016-06	1	1	1	0	0	0
2016-07	1	0	1	1	0	0
2016-08	1	1	1	0	0	0
2016-09	0	1	0	0	0	0
2016-10	1	1	0	0	1	0
2016-11	1	1	1	1	1	0
2016-12	1	1	0	1	0	0
2017-01	0	0	0	0	1	0
2017-02	0	0	1	0	0	0
2017-03	0	0	1	0	0	0
2017-04	1	1	1	1	1	0

2017-05	1	0	0	0	0	0
2017-06	0	0	1	0	0	0
2017-07	1	1	1	1	0	0
2017-08	0	0	1	0	0	0
2017-09	1	0	1	0	0	0
2017-10	0	1	0	1	0	0
2017-11	1	0	1	0	0	0
2017-12	0	1	0	0	0	0
2018-01	0	0	0	0	0	0
2018-02	0	0	0	0	0	0
2018-03	0	1	0	0	1	0
2018-04	0	0	0	0	0	0
2018-05	0	0	0	0	0	0
2018-06	0	0	0	0	0	0
2018-07	1	0	0	1	0	0
2018-08	1	0	0	1	0	0
2018-09	0	**0/P**	0	1	0	0
2018-10	1	**0/P**	0	0	0	0
2018-11	1	**1/P**	0	1	0	0
2018-12	1	**0/P**	0	0	0	0

Supplementary Table 4. List of peptides of milk-specific proteins identified from the orangutan fecal samples. “BLAST” shows the taxa for which the identical sequences were hit by the BLAST homology search.

Uniprot accession	Protein name	Start position	Sequence	Score	BLAST
H2NH48	Alpha-lactalbumin	25	CELSQLLK	199.07	Mammalia
		82	SSQVPQSR	143.61	Opisthokonta (mostly primates)
		90	NICDISCDK	121.58	Boreoeutheria
		90	NICDISCDKFLDDDITDDIMCAK	84.181	Simiiformes
		99	FLDDDITDDIMCAK	149.23	Simiiformes
		119	GIDYWLAHK	102.72	Eutheria
		128	ALCTEKLEQWLCEKL	78.814	Catarrhini
		134	LEQWLCEK	151.04	Mostly Boreoeutheria, but including other cellular organisms
H2PDG9	Alpha-S1-casein	99	CAEQFCR	149.06	Hominidae and bacteria
		106	LNEYNQLQLQAVHAQEQIHR	88.396	Catarrhini
H2PDH0	Beta-casein	39	VKHEDQQQGEDEHQNK	73.983	*Pongo*
		123	MPFFDPQMPK	163.15	*Pongo*
		133	LTDLENLHLPLPLLQPLMHQVPQPIPQTLALPR	139.82	*Pongo*
		166	QPLWSVPQPK	200.07	Catarrhini
		166	QPLWSVPQPKVLPIPQQVVPYPQR	83.063	Catarrhini
		176	VLPIPQQVVPYPQR	207.68	Catarrhini
H2PDH8	Kappa-casein	27	QPACHENDERPFYQK	191.78	Hominidae
		66	RPAIAINNPYVPHTYYANPAVVR	114.88	*Pongo*
		89	PHAQIPQQQYLPNSHPPTVER	140.16	*Pongo* and *Nomascus leucogenys*

Supplementary Table 5. List of peptides of lactase (Uniprot accession: H2P7G2) identified from the orangutan fecal samples.

Start positoin	Sequence	Score
267	LQTIEPK	102.71
276	VFIFNLK	80.377
453	FSISWSR	111.73
869	AFTFPSQVPSK	71.349
896	DLFYHGTFR	54.066
1085	DPGWAPYR	114.72
1104	VYHTYDEK	98.458
1176	SELQHLATSR	103.01
1186	LPSFTEEEKR	97.456
1199	ATADVFCLNTYYSR	176.42
1248	AVPWGTR	77.748
1294	TYINEALK	79.116
1354	YYTEVITNNGMPLAR	125.75
1369	EDEFLYGR	89.55
1432	IAEDLVTLQNLGVSHYR	73.279
1456	ILPDGTNR	91.9
1464	YISEAGLNYYVR	95.502
1517	EYADVLFQR	77.282
1607	DPSNQEDVEAAR	185.08
1660	LPEFTESEK	67.494
1724	MTPFGFR	103.28
1830	FYASVVR	99.919

Supplementary Table 6. List of biological defense proteins identified from the orangutan fecal samples.

Uniprot accession	Protein name	Organism	Gene name	Razor + unique peptides	Unique + razor sequence coverage (%)	Score
A0A2J8R8Y0	IGHM isoform 2 (Fragment)	*Pongo abelii*	CR201_G0052849	17	55.6	323.31
A0A2J8R8Y5	IGHG1 isoform 3 (Fragment)	*Pongo abelii*	CR201_G0052853	5	28.9	121.57
A0A2J8RA53	ELANE isoform 1	*Pongo abelii*	ELANE	6	34.1	14.612
A0A2J8RZ55	IGLV2-23 isoform 1 (Fragment)	*Pongo abelii*	CR201_G0047474	2	30.1	5.9887
A0A2J8RZ80	IGLV1-51 isoform 1 (Fragment)	*Pongo abelii*	CR201_G0047509	5	38.1	15.175
A0A2J8RZ87	IGLV1-50 isoform 1 (Fragment)	*Pongo abelii*	CR201_G0047507	4	48.5	29.792
A0A2J8SMN7	Ferritin	*Pongo abelii*	CR201_G0041585	3	31.9	3.7632
A0A2J8TFX5	Lactotransferrin	*Pongo abelii*	CR201_G0034903	40	63.1	323.31
A0A2J8TLQ5	SERPINA1 isoform 1	*Pongo abelii*	CR201_G0033988	9	23.2	76.724
A0A2J8W7V1	Lactoperoxidase	*Pongo abelii*	LPO	7	10.9	1.269
A0A2J8Y7S1	CST1 isoform 1	*Pongo abelii*	LOC100440716	10	66.4	323.31
A0A5E3XSY4	MHC class II protein	*Pongo pygmaeus*	Popy-DRB5	5	23.7	6.7269
A0A5E3XTB4	MHC class II protein	*Pongo pygmaeus*	Popy-DRB1	2	6.4	1.8433
A0A5E3XTI3	MHC class II protein	*Pongo abelii*	Poab-DRB5	2	8.6	1.864
A0A663DAW3	IGKC isoform 1 (Fragment)	*Pongo abelii*	CR201_G0053602	7	88.7	323.31
A0A6D2XP84	REG3A isoform 1	*Pongo abelii*	REG3A	7	41.7	141.96
A0A8I5TLQ5	Mammalian defensins domain-containing protein	*Pongo abelii*	LOC112134667	3	20.2	39.546
A4K2V7	SLPI isoform 1	*Pongo abelii*	SLPI	2	20.5	40.782
D8KYF7	MHC class II antigen (Fragment)	*Pongo pygmaeus*	Popy-DRA	2	9.4	3.3883
H2N5H8	Mucin-1	*Pongo abelii*	MUC1	2	10.1	40.509
H2N5P8	Protein S100	*Pongo abelii*	S100A9	8	82.5	323.31
H2NTI3	MPO isoform 1	*Pongo abelii*	MPO	10	19.7	9.8781
H2NTI5	EPX isoform 1	*Pongo abelii*	EPX	13	19.2	104.45
H2NWR5	PRTN3 isoform 2	*Pongo abelii*	PRTN3	4	26.2	5.8947
H2NX88	Complement C3	*Pongo abelii*	C3	14	11.4	11.877
H2P386	Trefoil factor 3	*Pongo abelii*	TFF3	6	45.7	178.51
H2P387	Trefoil factor 2	*Pongo abelii*	TFF2	4	50.4	10.917
H2P388	TFF1 isoform 1	*Pongo abelii*	TFF1	5	57.1	27.782
H2P3T5	Macrophage migration inhibitory factor	*Pongo abelii*	MIF	2	17.4	1.8333

H2PC99	Histidine-rich glycoprotein	*Pongo abelii*	HRG	6	16	114.16
H2PI73	BTN1A1 isoform 2	*Pongo abelii*	BTN1A1	10	21.7	44.206
H2PL15	MHC class II protein	*Pongo abelii*	LOC100456477	2	6.8	2.1324
H2PPG3	DEFA6 isoform 1	*Pongo abelii*	DEFA6	2	27	17.066
H2PRL6	Mammalian defensins domain-containing protein	*Pongo abelii*	LOC112131868	4	20.2	113.1
H2PTI2	Lipocalin/cytosolic fatty-acid binding domain-containing protein	*Pongo abelii*	LCN2	8	44.6	323.31
P79239	Lysozyme C	*Pongo pygmaeus*	LYZ	10	68.2	323.31
Q5R9L7	Serotransferrin	*Pongo abelii*	DKFZp459H0229	31	49.8	77.33
Q8HZE2	Beta-2-microglobulin (Fragment)	*Pongo pygmaeus*	–	2	33.3	4.7218
Q95426	MHC class I (Fragment)	*Pongo pygmaeus*	–	3	10.1	9.7843

Supplementary Table 7. Explanatory variables and intercept in linear mixed models for the normalized count of biological defense proteins.

Explanatory variables		Effect	SE	t	p-value
Fixed effect	**Intercept**	**0.1187**	**0.0078**	**15.266**	**<0.001**
	**Normalized milk-specific protein count**	**1.6783**	**0.5911**	**2.839**	**0.010**
	Age: Juvenile	-0.0202	0.0143	-1.405	0.174
	Age: Adolescent	0.0039	0.0122	0.322	0.750
	Sex: Male	-0.0093	0.0081	-1.155	0.260
Random effect	SD of Individuals	0.0002	0.0156	–	–

Supplementary Table 8. List of probiotic bacterial proteins identified from the orangutan fecal samples.

Uniprot accession	Protein name	Organism	Gene name	Razor + unique peptides	Unique + razor sequence coverage (%)	Score
A1A007	Small ribosomal subunit protein uS15	*Bifidobacterium adolescentis* (strain ATCC 15703 / DSM 20083 / NCTC 11814 / E194a)	rpsO	2	33.7	3.9434
A1A0S9	Small ribosomal subunit protein uS7	*Bifidobacterium adolescentis* (strain ATCC 15703 / DSM 20083 / NCTC 11814 / E194a)	rpsG	3	33.3	3.4008
A1A0T0	Elongation factor G	*Bifidobacterium adolescentis* (strain ATCC 15703 / DSM 20083 / NCTC 11814 / E194a)	fusA	9	16.4	19.439
A1A0T1	Elongation factor Tu	*Bifidobacterium adolescentis* (strain ATCC 15703 / DSM 20083 / NCTC 11814 / E194a)	tuf	9	27.3	55.048
A1A143	Enolase	*Bifidobacterium adolescentis* (strain ATCC 15703 / DSM 20083 / NCTC 11814 / E194a)	eno	6	18.1	10.243
A1A1N3	Phosphoglycerate kinase	*Bifidobacterium adolescentis* (strain ATCC 15703 / DSM 20083 / NCTC 11814 / E194a)	pgk	4	15.7	3.3205
A1A321	UPF0210 protein BAD_1323	*Bifidobacterium adolescentis* (strain ATCC 15703 / DSM 20083 / NCTC 11814 / E194a)	BAD_1323	2	4.4	1.8232
A1A3F7	Aspartyl/glutamyl-tRNA(Asn/Gln) amidotransferase subunit C	*Bifidobacterium adolescentis* (strain ATCC 15703 / DSM 20083 / NCTC 11814 / E194a)	gatC	4	40.8	6.05
B8DUT6	Probable phosphoketolase	*Bifidobacterium animalis* subsp. lactis (strain AD011)	BLA_1483	4	6.2	20.558
B8DVY5	Large ribosomal subunit protein bL12	*Bifidobacterium animalis* subsp. lactis (strain AD011)	rplL	2	19	4.0037
B8DW17	Small ribosomal subunit protein uS19	*Bifidobacterium animalis* subsp. lactis (strain AD011)	rpsS	2	34.8	3.0011
O32755	Glyceraldehyde-3-phosphate dehydrogenase	*Lactobacillus delbrueckii* subsp. bulgaricus	gap	2	6.5	3.6515
P17615	DNA-binding protein HB1	*Bifidobacterium longum* (strain NCC 2705)	hup	2	18.3	28.947
P59159	2,3-bisphosphoglycerate-dependent phosphoglycerate mutase	*Bifidobacterium longum* (strain NCC 2705)	gpmA	3	17.1	4.6084
Q38YF8	S-adenosylmethionine synthase	*Latilactobacillus sakei* subsp. sakei (strain 23K)	metK	4	9.3	5.2139
Q74L85	Small ribosomal subunit protein uS19	*Lactobacillus johnsonii* (strain CNCM I-12250 / La1 / NCC 533)	rpsS	4	34.7	1.6812
Q8G444	Large ribosomal subunit protein uL10	*Bifidobacterium longum* (strain NCC 2705)	rplJ	2	14.5	3.5333
Q8G574	Pyridoxal 5-phosphate synthase subunit PdxS	*Bifidobacterium longum* (strain NCC 2705)	pdxS	3	12.7	16.51
Q8G6V2	Ketol-acid reductoisomerase (NADP(+)) 1	*Bifidobacterium longum* (strain NCC 2705)	ilvC1	3	13.1	4.2729
Q8G756	Small ribosomal subunit protein bS6	*Bifidobacterium longum* (strain NCC 2705)	rpsF	2	22.7	2.1006

Q8G7I6	Glucose-6-phosphate isomerase	*Bifidobacterium longum* (strain NCC 2705)	pgi	4	9.9	9.8288
Q8KML6	Chaperone protein DnaK	*Fructilactobacillus sanfranciscensis*	dnaK	3	4.9	1.5208
Q9CDX0	Large ribosomal subunit protein uL29	*Lactococcus lactis* subsp. lactis (strain IL1403)	rpmC	2	26.1	1.4548
Q9CHE9	UPF0337 protein YhjA	*Lactococcus lactis* subsp. lactis (strain IL1403)	yhjA	4	50.6	6.3466
Q9CI64	DNA-binding protein HU	*Lactococcus lactis* subsp. lactis (strain IL1403)	hup	4	50.5	9.435

Supplementary Table 9. Explanatory variables and intercept in linear mixed models for the normalized count of probiotic bacterial proteins.

Explanatory variables		Effect	SE	t	p-value
Fixed effect	**Intercept**	**0.0442**	**0.0191**	**2.313**	**0.031**
	**Normalized milk-specific protein count**	**3.4878**	**1.4525**	**2.401**	**0.025**
	Age: Juvenile	-0.0072	0.0299	-0.240	0.812
	Age: Adolescent	-0.0604	0.0352	-1.715	0.100
	Sex: Male	-0.0098	0.0198	-0.495	0.626
Random effect	SD of Individuals	0.0015	0.0382	–	–

Supplementary Table 10. List of pathology-related bacterial proteins identified from the orangutan fecal samples. Note that “Organism” is representative of the species contained in the database, but the same sequences are also found in species that are not included in the database. Therefore, the species shown cannot be uniquely determined (see Table 11).

Uniprot accession	Protein name	Organism	Gene name	Razor + unique peptides	Unique + razor sequence coverage (%)	Score
P80583	Flagellin (Fragment)	*Clostridium tyrobutyricum*	fla	3	10.9	323.31
P38507	Immunoglobulin G-binding protein A	*Staphylococcus aureus*	spa	2	7.9	108.25
P13713	Flagellin	*Serratia marcescens*	fliC	2	5.1	5.6435
Q06983	Flagellin	*Salmonella senftenberg*	fliC	3	7.5	4.4249

Supplementary Table 11. List of peptides of pathology-related proteins identified from the orangutan fecal samples. “BLAST” shows the taxa for which the identical sequences were hit by the BLAST homology search.

Uniprot accession	Protein name	Start position	Sequence	Score	BLAST
P13713	Flagellin	271	ALAQVDGLR	59.205	Bacteria
P13713		280	SSLGAVQNR	147.16	Bacteria
P38507	Immunoglobulin G-binding protein A	103	DQQSAFYEILNMPNLNEEQR	141	*Staphylococcus*
P38507		161	EQQNAFYEILNMPNLNEEQR	153.36	Staphylococcaceae
P80583	Flagellin (Fragment)	39	AGDDAAGLAISEK	257.8	Bacteria
P80583		315	LGAYQNR	114.67	Cellular organisms
P80583		322	LEHTINNLGTSSENLTSAESR	62.891	Bacteria
Q06983	Flagellin	43	DDAAGQAIANR	67.897	Gammaproteobacteria
Q06983		346	TKNESAKLSDLEANNAVK	48.741	*Salmonella*
Q06983		434	SSLGAIQNR	142.64	Bacteria

Supplementary Table 12. Information of the subject orangutan individuals.

Name	N. of samples	Age class	Sex	First observation date	Mother
Danum	5	juvenile	F	2011-03-02	Sheena
Kate	6	juvenile	F	2012-07-15	Lina
Lom	4	juvenile	M	2010-06-06	Beth
Lya	2	juvenile	F	2014-02-09	Linda
Sely	3	juvenile	F	2011-06-05	Yanti
Kimi	1	adolescent	M	2009-03-15	Sumi
Yamato	2	adolescent	F	2005-09-22	Sumi
Lina	2	adult	F	2004-07-15	–
Linda	1	adult	F	2005-09-14	–
Sheena	1	adult	F	2004-08-23	–

Supplementary Data

Supplementary Data 1 (separate file). List of the identified protein groups from the orangutan fecal samples. Also uploaded to Zenodo (*3*).

Supplementary Data 2 (separate file). List of the identified protein groups from the orangutan milk samples reported in Cleland and Power (*4*). Also uploaded to Zenodo (*3*).

Supplementary References

1. S. Safika, et al., First Study on profiling of gut microbiome in wild and captive Sumatran orangutans (*Pongo abelii*). *Vet World* 16, 717–27 (2023).
2. T. Tsutaya, et al., Stable isotopic investigation of the feeding ecology of wild Bornean orangutans. *Am J Biol Anthropol* 179, 276–90 (2022).
3. T. Tsutaya, Fecal proteomics of immature wild Bornean orangutans. *Zenodo* https://doi.org/10.5281/zenodo.17309234 (2025).
4. T. P. Cleland, M. L. Power, Variation in Milk Proteins Across Lactation in *Pongo pygmaeus* and *Gorilla gorilla. J Proteome Res* **21**, 2647–2654 (2022).
