# Supplementary material for: Continuous and prolonged breastfeeding in wild Bornean orangutans verified with fecal proteomics
Source: Commun Biol. 2026 May 25;9:973. doi: 10.1038/s42003-026-09968-2 (PMC13376732; doi:10.1038/s42003-026-09968-2)
Supplement: Supplementary file 2 — Description of Additional Supplementary Materials [file 42003_2026_9968_MOESM2_ESM.pdf]

Description of Additional Supplementary Files

File name: Supplementary Data 1

Description: List of the identified protein groups from the orangutan fecal samples. Also uploaded to Zenodo (https://doi.org/10.5281/zenodo.17309234).

File name: Supplementary Data 2

Description: List of the identified protein groups from the orangutan milk samples reported in Cleland and Power (2022). Also uploaded to Zenodo (https://doi.org/10.5281/zenodo.17309234).
