# Supplementary material for: Continuous and prolonged breastfeeding in wild Bornean orangutans verified with fecal proteomics
Source: Commun Biol. 2026 May 25;9:973. doi: 10.1038/s42003-026-09968-2 (PMC13376732; doi:10.1038/s42003-026-09968-2)
Supplement: Supplementary file 5 — Reporting Summary [file 42003_2026_9968_MOESM5_ESM.pdf]

nature portfolio

Corresponding author(s): Takumi Tsutaya

Last updated by author(s): Oct 10, 2025

Reporting Summary

Nature Portfolio wishes to improve the reproducibility of the work that we publish. This form provides structure for consistency and transparency in reporting. For further information on Nature Portfolio policies, see our Editorial Policies and the Editorial Policy Checklist.

Statistics

For all statistical analyses, confirm that the following items are present in the figure legend, table legend, main text, or Methods section.

n/a	Confirmed	
☐	☒	The exact sample size (*n*) for each experimental group/condition, given as a discrete number and unit of measurement
☐	☒	A statement on whether measurements were taken from distinct samples or whether the same sample was measured repeatedly
☐	☒	The statistical test(s) used AND whether they are one- or two-sided<br>*Only common tests should be described solely by name; describe more complex techniques in the Methods section.*
☐	☒	A description of all covariates tested
☒	☐	A description of any assumptions or corrections, such as tests of normality and adjustment for multiple comparisons
☐	☒	A full description of the statistical parameters including central tendency (e.g. means) or other basic estimates (e.g. regression coefficient) AND variation (e.g. standard deviation) or associated estimates of uncertainty (e.g. confidence intervals)
☐	☒	For null hypothesis testing, the test statistic (e.g. *F*, *t*, *r*) with confidence intervals, effect sizes, degrees of freedom and *P* value noted<br>*Give P values as exact values whenever suitable.*
☒	☐	For Bayesian analysis, information on the choice of priors and Markov chain Monte Carlo settings
☒	☐	For hierarchical and complex designs, identification of the appropriate level for tests and full reporting of outcomes
☒	☐	Estimates of effect sizes (e.g. Cohen's *d*, Pearson's *r*), indicating how they were calculated

Our web collection on statistics for biologists contains articles on many of the points above.

Software and code

Policy information about availability of computer code

Data collection	pFind and MaxQuant to obtain protein identification results; Proteomic data were uploaded to the PRIDE repository with an identifier PXD060699
Data analysis	R to process data; Scripts were uploaded to the Zenodo repository with the digital object identifier 10.5281/zenodo.17309234

For manuscripts utilizing custom algorithms or software that are central to the research but not yet described in published literature, software must be made available to editors and reviewers. We strongly encourage code deposition in a community repository (e.g. GitHub). See the Nature Portfolio guidelines for submitting code & software for further information.

Data

Policy information about availability of data

All manuscripts must include a data availability statement. This statement should provide the following information, where applicable:

- Accession codes, unique identifiers, or web links for publicly available datasets
- A description of any restrictions on data availability
- For clinical datasets or third party data, please ensure that the statement adheres to our policy

Proteomic data were uploaded to the PRIDE repository with an identifier PXD060699. R scripts used to analyze data have been uploaded to the Zenodo repository with the digital object identifier 10.5281/zenodo.17309234. Other data is reported in the Supplementary Information.

Research involving human participants, their data, or biological material

Policy information about studies with human participants or human data. See also policy information about sex, gender (identity/presentation), and sexual orientation and race, ethnicity and racism.

Reporting on sex and gender	n/a
Reporting on race, ethnicity, or other socially relevant groupings	n/a
Population characteristics	n/a
Recruitment	n/a
Ethics oversight	n/a

Note that full information on the approval of the study protocol must also be provided in the manuscript.

Field-specific reporting

Please select the one below that is the best fit for your research. If you are not sure, read the appropriate sections before making your selection.

☒ Life sciences ☐ Behavioural & social sciences ☐ Ecological, evolutionary & environmental sciences

For a reference copy of the document with all sections, see nature.com/documents/nr-reporting-summary-flat.pdf

Life sciences study design

All studies must disclose on these points even when the disclosure is negative.

Sample size	All samples collected during the 31 months of fieldwork have been used.
Data exclusions	No data was excluded.
Replication	No replication was done.
Randomization	Sample collection or the encounter into each orangutan individual are random.
Blinding	No blinding was applied.

Reporting for specific materials, systems and methods

We require information from authors about some types of materials, experimental systems and methods used in many studies. Here, indicate whether each material, system or method listed is relevant to your study. If you are not sure if a list item applies to your research, read the appropriate section before selecting a response.

Materials & experimental systems

n/a	Involved in the study	
☒	☐	Antibodies
☒	☐	Eukaryotic cell lines
☒	☐	Palaeontology and archaeology
☐	☒	Animals and other organisms
☒	☐	Clinical data
☒	☐	Dual use research of concern
☒	☐	Plants

Methods

n/a	Involved in the study	
☒	☐	ChIP-seq
☒	☐	Flow cytometry
☒	☐	MRI-based neuroimaging

Animals and other research organisms

Policy information about studies involving animals; ARRIVE guidelines recommended for reporting animal research, and Sex and Gender in Research

Laboratory animals	n/a
Wild animals	Free-ranging wild Bornean orangutans (Pongo pygmaeus) were the subject of this study. No intervention was performed. No invasive sample collection was performed.
Reporting on sex	Both sexes are included.
Field-collected samples	Samples were kept in a freezer until the analysis.
Ethics oversight	This research was approved by the Sabah Biodiversity Centre (SaBC) and Danum Valley Management Committee, with the license reference numbers JKM/MBS.1000-2/2 JLD.4 (87), JKM/MBS.1000-2/2 JLD.5 (150), and JKM/MBS.1000-2/2 JLD.15 (17). The sample transfer was approved by SaBC and the Sabah Wildlife Department with the license reference numbers JKM/MBS.1000-2/3 JLD.3 (46) and JKM/MBS.1000-2/3 JLD.5 (19). All procedures of the research followed the guideline "Code of Best Practices for Field Primatology" and were approved by the Ethics Committee for Animal Research of the Graduate University for Advanced Studies (SKD2024AR006).

Note that full information on the approval of the study protocol must also be provided in the manuscript.

Plants

Seed stocks	n/a
Novel plant genotypes	n/a
Authentication	n/a
