# Supplementary material for: Continuous and prolonged breastfeeding in wild Bornean orangutans verified with fecal proteomics
Source: Commun Biol. 2026 May 25;9:973. doi: 10.1038/s42003-026-09968-2 (PMC13376732; doi:10.1038/s42003-026-09968-2)
Supplement: Supplementary file 6 — Transparent Peer Review file [file 42003_2026_9968_MOESM6_ESM.pdf]

nature portfolio

Peer Review File

Continuous and prolonged breastfeeding in wild Bornean orangutans verified with fecal proteomics

Corresponding Author: Dr Takumi Tsutaya

This file contains all reviewer reports in order by version, followed by all author rebuttals in order by version.

Version 0:

Reviewer comments:

Reviewer #1

(Remarks to the Author)
This study uses fecal proteomics to detect milk protein signatures in fecal samples from wild Bornean orangutans. Fecal samples from juvenile, adolescent, and adult orangutans were analyzed and compared to descriptions of milk proteins in orangutan and other species' milk. Milk proteins (and lactase) were found in all feces from juveniles under 7 years of age, but not in any feces of orangutans older than 7 years. The patterns in milk proteins in juvenile feces confirms nutritive suckling to around 6.5 years of age. This result contrasts with results from previous techniques which showed either minimal breastfeeding after 2.7 years of age or possible "cyclical" breastfeeding by older juveniles. The data are convincing and important to publish.

2. Overall impression of the work:

The study and experimental design in and of itself is worthy of publication. The paper presents convincing evidence that the technique of using fecal proteomics to detect breastfeeding using feces is a viable and valuable tool. In general, the discussion of orang life history and lactation strategy is useful, but there is a marked reliance on the k-selection theory, an outdated life history theory, to explain orangutan parental investment. The paper would be improved if the authors would be more precise in what they are investigating. This isn't about life history in general. It is about the potential adaptive significance of extended lactation in orangs. Lactation period indicates much more than time between offspring and is an incredibly energetically taxing process that in most species far exceeds gestation in energy required. Tracking weaning periods for offspring can tell us about the parental care needed for offspring success as well as how it is changing with orangutan habitat degradation, parental age, etc.

The effort to frame the importance of the results around lactation related infertility or amenorrhea and extended interbirth intervals is overstated. Lactation related amenorrhea is a consequence of lactation in most primates (not the callitrichid primates), but it is a stretch to state that the natural consequence is somehow an adaptive feature that was under selection. It wasn't selected against, but the selective advantage more likely is benefits to the juvenile. The longer interbirth interval is a consequence that was not selected against. This study provides no data that selection acted to increase the interbirth interval; just that it wasn't selected against.

The study itself is worthy of publication and presents novel methodologies and techniques for understanding parental care and lactation in orangutans. With some framework revisions this will be an excellent paper.

3. Specific comments with recommendations:

There is no need to bring in "k-selection theory" which is an old (somewhat still useful) framework for looking at life history. The first line of the abstract says what is important: "Orangutans have a slow but secure life history strategy." [Please leave out words like "extremely".] Orangs have the longest interbirth intervals among primates, with low infant mortality. They appear to have extended lactation, with breastfeeding of juveniles extending longer than other great apes.
Those are the important factors that this study examines, and, for the extended breastfeeding claim, actually tests. The authors are urged to be precise, and not wander into statements like: Lines 190-191; "Contrary to chimpanzees and gorillas, humans are characterized by a slow and secure life history, similar to orangutans." Many would argue with humans having a "slow" life history relative to chimps and gorillas, given the shorter interbirth intervals of humans. What is true is that human

and orang offspring have a longer period of time where they are dependent on adults. For orangs, juveniles are dependent on their mother, receiving breastmilk as an important resource. For humans, the cooperative childcare system allows longer "dependency". These are interesting points to pursue in this paper that do not need to be disguised as "life history". Life history strategy is bigger than that. Be precise in what you are discussing and do not wander into jargon and theory.

The title is broader than the study warrants. A suggested title would be: "Fecal proteomics as a novel technique to verify continuous and prolonged breastfeeding in the orangutan." The focus of the study is really on proof of extended lactation and less on life history theory in general.

The last sentence of the Abstract is excellent (Lines 52-54). The sentence before is a stretch (Lines 49-51). It is fine to bring in some discussion of human lactation/reproductive strategy in the Discussion, but the paper does not "highlight" the uniqueness of humans. Also, the data presented here do not demonstrate that extended breastfeeding "provides" the physiological and molecular basis for the slow life history of orangutans [again, avoid words like "unique"; this paper does not address all known mammals; and there are plenty out there we do not know much about]. Fair to say that extended lactation is a key component of the slow life history of orangutans. But do we know that it is lactational amenorrhea that stops females from cycling or is it amenorrhea related to low body condition? The extended lactation likely contributes to female low body condition, as it probably is a significant cost, but seasonal food availability would also contribute. Again, fair for the authors to hypothesize that lactation (i.e. the hormonal milieu of lactation) contributes to amenorrhea and a long interbirth interval, but they haven't shown that it is the only or even the most important factor.

The last sentence of the first paragraph of the Introduction (Lines 98-100) detracts rather than adds to the paper. There is no reason to bring in "K-strategy". Focus on what the paper investigates. Do orangutans have an extended lactation that would nutritionally benefit older juveniles? At what age does nutritive suckling appear to end?

Delete the first sentence of the second paragraph of the Introduction for the same reason. Edit the first sentence of the third paragraph to take out K-selection and the word "extremely". Words like extremely are value judgements and should be used rarely in scientific publications. Better sentence would be "We hypothesize that prolonged periods of breast milk intake, which provide enhanced biological defense agents in juveniles and contributes to the long interbirth interval for adult females, underly the success of the slow life history of orangutans." Lead with what you are studying and what you demonstrate in your results.

The last paragraph of the Introduction is excellent. This is what your paper is showing.

The Results are clear and easy to follow. Congratulations to the authors for producing interesting and informative data.

Specific line by line comments and suggestions:
61-65: States that lactation "determines" the reproductive speed and strategy. Determines should not be used in this context, lactation does not necessarily "determine" anything and the reproductive speed can be affected by a myriad of factors, including birth of altricial young. What "determines" speed is not addressed in this paper so should be reframed. Lactation is certainly connected to a slow reproductive speed but what determines what is uncertain until explicitly tested.
63: "breastfeeding period and older age at the end of weaning represent the so-called "K strategy," which is characterized by a small number of litters, slow growth, and a long 65 life span (1)." Remove "so-called "K-strategy" and replace with slow life history, use of k-strategy distracts from novel findings.
66-67: "An extreme K-strategy may be considered the slow but secure life history of orangutans (5, 6)." Suggest that this sentence simply be deleted. Could be reworked to say "Orangutans have a slow but secure life history characterized by longest interbirth intervals among primates, with low infant mortality, associated with an extended lactation".
78-79: Delete "k-strategic" and utilize slow life history instead.
79-80: "include prolonged periods of breast milk intake, an increase in infant biological defense agents, and maternal interbirth intervals" Replace with "include prolonged periods of breast milk intake, an increase in infant biological defense agents, and prolonged maternal interbirth intervals". Sentence is not grammatically correct.
163-164: "Bifidobacterium dominates the intestine of healthy breastfed infants and improves their health (26)." Please specify human infants here.
176-177: Remove "k-selection" and "extremely"
181-183: "The fact that the mothers of breastfed juveniles were not pregnant with their next sibling indicates that long breastfeeding periods are closely related to long birth intervals (3, 4), even in orangutans." Many animals get pregnant or begin the gestation process during lactation with phenomena such as delayed implantation and sperm storage. It is also generally unnecessary for the paper to emphasize long interbirth intervals as an ultimate cause as it may be a proximate cause, due to effects from maternal body condition, environmental constraints, etc. It is also difficult to argue that it is an evolved trait.
185-186: "which facilitates the health of immature orangutans." Instead say "which likely facilitates the health of immature orangutans" as this was not tested in this study.
189-190: The real point of this paragraph is that human and orangutan juveniles become competent and independent later than chimpanzee and gorilla offspring and rely on adults. Please reword paragraph.
198-199: "This study emphasized the two contrasting evolutionary foundations for the similar, "convergent" slow life histories that occur in hominid species." No it does not. Remove this sentence.
209: This may just mean that proportion of nutrient needs met from milk is much lower after 2.7 years. The stable isotope data indicates that the majority of nutrition comes from milk before age 2.7 years. Combined with the novel and important findings from this study the implication is that while milk continues to be ingested after age 2.7 years of age, milk no longer contributes the majority of nutrition for juveniles. Rather, the same foods that the mother eats likely is contributing most of the

nutrition. Hence, no measurable difference in stable isotope ratios.
214-218: Similarly, the “cyclical” breastfeeding findings could arise from the probable wide variation in the proportion of nutrient ingestion over seasons from milk versus wild foods. If you assume that milk intake will remain roughly equal over the last few years of lactation, then seasonal changes in other food intake would drive the “signal” for both stable isotopes, as discussed above, and for calcium-normalized barium concentrations laid down in tooth enamel. The authors’ data combined with the previous findings build a nice picture of breast milk contributing a variable proportion of nutrition after 2.7 years of age, probably driven by seasonal food supply. The findings are consistent with milk providing a relatively constant amount of nutrients for juveniles, with the juveniles obtaining substantial nutrition from the same foods as the mother, but with large seasonal variation.
225-226: “intervals of several months (3), and no other mammals have been reported to exhibit such cyclical breastfeeding patterns (1).” This is not necessarily true, fur seals and sea lions leave their offspring and stop producing milk, returning to lactate after periods of days and even weeks, this can be said for primates but not for all mammals. Please reword and say “…no other primates have yet been reported to exhibit…”. There is still much we do not know about many mammals’ lactation so there is always the possibility that there are primates who have cyclical breastfeeding patterns. However, the mechanism given above for producing the same results in stable isotope and tooth enamel barium is far more likely. Those methods rely on a large proportion of nutrition coming from milk. When large amounts of nutrients come into the system from other foods the signal for milk intake will be lost in the noise.
233-235: “The results of this study suggest this low reproductive rate partly originates from the physiological and molecular traits of prolonged breastfeeding periods of orangutans.” Lactation has not been proven, in this paper or others, to be the ultimate mechanism for low reproductive rate, please remove “partly originates”. This sentence can be rephrased to say “The results of this study confirm that orangutans have prolonged breastfeeding periods and maternal investment, which likely contribute to both the high juvenile survival and low adult female reproductive rate.”
236: “… interventions that shorten the breastfeeding period in captive or wild orangutans may increase the population by ceasing lactational amenorrhea earlier and shortening the interbirth interval (3, 4); however, such interventions would be accompanied by long term negative effects by either completely or partially undermining the short-term and lifelong health benefits of breast milk intake (2).” While this may be an important takeaway from the study, it should be framed as a speculation and not as a main takeaway. Also, in Shumaker et al 2008 (https://doi.org/10.1159/000137705) the authors note that in association with the earlier age at menarche and shorter interbirth intervals of captive orangs there is also menopause at about age 41. Something not seen in the wild. Anthropogenic interventions often have unexpected consequences.

The end of the paper should emphasize the confirmation of active lactation period using novel techniques first and then the varying implications (including the one listed here). Emphasizing that the data points to longer lactation periods for reproductive success and offspring survival is essential.

Michael L. Power, PhD
Animal Scientist
Center for Species Survival
Smithsonian's National Zoo and Conservation Biology Institute

Reviewer #2

(Remarks to the Author)
This MS reports on an amazing amount of detailed work and shows how proteomics can be applied to non-invasively collected fecal samples from natural population of wild mammals. This method yields detailed milk-intake-specific results and is a huge improvement over other methods recently used to estimate milk intake in great apes in the wild. Yet, this message deserves more prominence in the title and framing of the MS and I hope the feedback is useful to do that.

Strong point: Proteomics applied for the first time to detect maternal milk intake by immatures in a natural population of orangutans. Despite the current small sample size this approach convincingly documents actual milk consumption by the offspring over almost the complete interval of many (here > 6.5) years before a younger sibling is conceived. The results take away any doubts about the frequently reportedly exceptionally long period of offspring dependence and consequently long inter-birth intervals. In contrast, other recent studies on primates to detect milk intake using isotopes could not separate milk from other animal-based sources of isotopes and thus yielded inconclusive results and dubious interpretations (e.g. Badescue et al. 2017; Smith et al. 2017). Thus, this study is an important contribution to refute such assumptions about ‘comfort suckling’ or ‘cyclical lactation’ and the results fully concur with extensive observational data on numerous wild populations.

Weaker point: Even though the abstract is clear, it is not immediately obvious from the title and framing of the introduction that and how proteomic analyses are a great contribution to our understanding of the multi-year lactation pattern of great apes as well as other mammals with slow life history. The slow life history of orangutans is prominent in the title and introduction but was already sufficiently documented in the literature (as also cited by the authors). There is no need to discuss K-strategies etc. mentioning slow life history should suffice to direct the attention to the lactation pattern. However, in my view the MS should immediately draw attention to the advantage of a better documentation of the long and consistent maternal investment through lactation. In the discussion this aspect of the results could also be addressed more clearly and be discussed in relation to the sustained maternal effort (based on time budgets) over most of the long lactation and interbirth period as reported by van Noordwijk et al. 2013 DOI 10.1007/s00265-013-1504-y; and van Noordwijk, Kuzawa & van Schaik 2013 DOI: 10.1002/evan.21368.

General comments

Terminology: infants, juveniles and adolescents

In animal studies the term 'juvenile' is generally used for immatures, which are expected to survive the death of their mother/caretaker but are not yet sexually mature (e.g. Pereira 1993 In Fairbanks & Pereira 1993*). Despite growing evidence offspring survival is not exclusively dependent on weaning stage, it is common practice to use 'infant' for pre-weaned offspring (still receiving milk) and 'juvenile' for fully weaned immatures. Alternatively, the terms dependent vs. independent (or weaned) immatures have been used esp. for orangutans. The term adolescent is used for the developmental stage with rapid hormonal changes into sexual maturity. Without hormonal analyses the onset of this tends to be hard to recognize in wild primates but is usually considered to start multiple years after weaning in wild orangutans. The age classification used in this MS seem to be based on the faster growth of orangutans in captivity/human-provisioned situations. However, since actual ages of the immatures are known, there is no need to use any of these terms and instead use ages and conclude based on the results whether which individual was still ingesting milk and thus pre-weaned and which individuals were fully weaned. For weaned individuals the only relevant further classification would be whether they have already reached adult body size.

• Pereira, M.E. and Fairbanks, L.A. eds., 1993/2002. Juvenile primates: life history, development and behavior, with a new foreword. University of Chicago Press.

Breast milk: this term is typically used for humans to distinguish it from milk from other sources (animals, plants or industrial infant formulas). For wild mammals, milk is always maternal milk (in rare cases from allomothers) so can just be referred to as milk.

Other comments

Line 69 Confusing sentence: currently orangutans only occur in Sumatra and Borneo however the 3 spp. are separated in distribution 2 in Sum 1 in Borneo. Maybe mention the 3 spp. in previous sentence for easier flow?

Line 73-77 these details seem not relevant for this MS

Line 72 change to suckling from the mother's nipple

Line 77 most human populations

Line 81-82 This is the main topic of this paper! And deserves more prominence….

Line 83-4 comfort suckling is not observed but assumed… if one cannot easily see whether milk is transferred comfort suckling is an interpretation based on lack of evidence of actual transfer…. In fact, the current study could show whether 'comfort suckling' happens over an extended period in this orangutan population by combining observations suckling with evidence for milk-consumption. (In fact, this study shows that comfort suckling is certainly not happening over a long period…)

Line 86-9 fully agree! See also Badescu et al. 2025 DOI: 10.1002/ajp.23552 for isotope results based on hair suggesting longer milk intake than previously concluded based on fecal isotopes.

Line 91 Pongo pygmaeus morio: add subspecies. This is relevant since the morio subspecies was long thought to have shorter interbirth intervals than the other subspecies or Sumatran orangutans (even though based on small sample sizes van Noordwijk et al. 2018: reference 6) did not find a consistent difference).

Line 118 Figure 1 shows variation in the presence of actual milk-specific proteins for all dependent immatures without a clear age effect. This seems consistent with a rather steady milk intake over many years in line with the sustained maternal effort (fluctuating with fruit abundance but not offspring age) over most of the long lactation period reported by van Noordwijk et al. 2013a&b. (see doi above). Even though their study was on a different population of orangutans, it would be appropriate to discuss these results in relation to their results on maternal effort (feeding time) over the course of the long interbirth intervals of orangutans.

Line 179 please mention that ref 20 is based on the same population which makes the consistency as strong as possible. Of course it is also consistent with other populations….

Line 180 suggests that Danum Valley may be exceptional even among orangutans, which it is not (in this respect): for all populations included in van Noordwijk et al. 2018 contributors also mention suckling until shortly before or even after the mother conceived a subsequent offspring. With an overall average of 7.6 years between births, many orangutans suckle for more than 7 years. It's not clear how relevant human reference 3&4 are here.

Line 190 Explain what you mean by secure life history? Low infant mortality?

Line 198-199 What is meant by 'two contrasting evolutionary foundations for the similar, "convergent" slow life histories that occur in hominid species.' All great apes have the same foundation of multi-year lactation with a long period of overlap between increasing ingestion of solid food with a consistent buffer of maternal milk. Yet humans managed to reduce the period of offspring milk-dependence despite their increased requirements due to larger brain size) thanks to active food processing (cutting, mashing, cooking etc.) as well as active provisioning and alloparents. (e.g. van Noordwijk Kuzawa van Schaik 2013; van Schaik 2016 The Primate Origins of Human Nature, Wiley& Sons).

Line 200-230 Yes! proteomics is the more reliable tool to assess milk-intake. Previous studies based on isotopes in fecal matter and teeth have lead to confusing and premature conclusions, probably because these studies did not take actual food intake and age-dependent digestibility of solid food into account. Your results should restore trust in long term behavioral observations even when some lab-based analyses lead to unlikely conclusions. Thank you!

Line 236-240 Such manipulation of the natural reproductive pattern is known for captivity and 'rehabilitation' projects but should have no place in conservation efforts of a species in its natural habitat and does not respect orangutans' complex social organization. The suggestions might increase the number of orangutans alive (but see negative consequences such as higher infant mortality under the proposed circumstances reported by Kuze et al. 2012 (DOI 10.1007/s10329-011-0285-z) and Galdikas & Ashbury 2012 (DOI 10.1007/s10329-012-0331-5). However, such manipulation would not increase any natural and healthy population! Currently, the main problem for orangutan conservation is a lack of large stretches of safe habitat where orangutans can maintain their own lifestyle. The main message for conservation from this paper is that existing populations need protection because their slow life history implies they can hardly recover from any population crashes…

References
I respect that references often reflect a personal history and preference of the authors, nevertheless in many cases alternative references may be more appropriate e.g.

Ref 1: See Lee 1996 in Evol Anthro: https://doi.org/10.1002/(SICI)1520-6505(1996)5:3<87::AID-EVAN4>3.0.CO;2-T

Ref 4 Bongaarts' parameters seem chosen to compare human populations – it would be more relevant to refer to primate literature here. Specifically, orangutan literature with insights into their social organization which is very different from human conditions. At least the need for an extended period of mating to allow for meeting and mating with many different males is relevant here e.g. Knott et al. 2010 doi: 10.1098/rspb.2009.1552 ; Kunz et al. 2021 https://doi.org/10.1016/j.anbehav.2021.09.012 ; Kunz et al. 2023DOI: 10.1002/ajp.23535 ; Scott et al. 2019 https://doi.org/10.1007/s10764-019-00097-8 ; van Noordwijk et al. 2023 https://doi.org/10.1007/s00265-023-03407-6

Ref 16 Miller et al . on human seems not the most relevant reference to make the point about unreliable observation for primates due to missing overnight or high in canopy suckling… see e.g. Badescu et al. 2017; van Noordwijk et al. 2013

Figures
Fig 1 vs. Fig S1 explain the difference between normalized intensity and normalized counts?
Fig 2 please label the y-axis
Fig 3B: x-axis label needs fixing at right end 'Adult'
Fig S3-6 please provide legend explaining the graphs including the meaning of the colors
Fig S8 label y-axes as probiotic bacterial proteins

Reviewer #3

(Remarks to the Author)
The authors use fecal proteomics on a wild population of orangutans to identify the timing of weaning across 10 individuals of various ages. They also identify the number of dietary and potentially probiotic bacterial proteins, which they associate with the presence of milk in the juvenile diet. I think this is an outstanding paper that uses a novel technique to address an interesting important ecological question. It is also an advancement over previous fecal proteomics research in that it extends the method to a free-ranging population opening up many possibilities for molecular ecological research. That said I have many questions about the data analysis that should be addressed and clarified in the paper where appropriate.

Sampling:

-There is uneven sampling among the age and sex categories. For instance, all non-juvenile samples are female. Can you justify why this does not affect your results? If you run the same analyses with only female juveniles, do you get comparable results? Also, you have a variable number of samples per individual. While this shouldn't affect the broader result of milk proteins being absent in adolescents and adults, it could affect your linear models. It would also helk the reader to include a supplemental table with the number of samples per individual.

Proteomics database searches:

-It is difficult for the reader to assess what is in the protein databases. SwissProt is a large database, but the reader doesn't really know what you are searching against. For example, how many plants bacteria, etc? Please provide a supplemental table that describes the number of major taxa.

-Please also provide a supplemental table that lists the number of proteins in the databases that you are assigning to each category you each of your investigated categories. For example, how many milk proteins are there in the database (not just the number you found)? How many biological defense proteins, etc?

-Why is SwisProt the appropriate choice for the database? Other metaproteomic studies have opted for broader initial databases (the entire UniProt, NCBI, databases) or more targeted choices (selecting only the microbes found in microbiome studies of the same species).

-Why do you use pFind for the first database search and MaxQuant for the second? Many different database search tools are used in proteomics and metaproteomics. It is not clear to me why MaxQuant should be considered more accurate (or appropriate for the second search) than pFind or other programs.

-Why do you set the threshold for 2+ razor-unique peptides in the second database search. If a single peptide is identified with high confidence, isn't the probability of if not being correct is extraordinarily low, even in the context of a large database? This seems like an unnecessarily strict threshold.

-How do you address the presence of multiple isoforms in your database? Proteomes often include multiple isoforms of the same protein which can bias your searches.

-Why did you include proteomes from multiple species of orangutans when you only have one species in your database? I would think it makes sense to just use your target species.

-What is the source of the contaminants database?

-Please clarify why you use an FDR of 1% for the peptides and 100% for the proteins in the initial database?

-Please justify your choice of fixed and variable modifications. Are these choices made based on the type of sample, extraction method or something else?

Statistics

- The underlying assumption about your normalization approach as described in the supplement makes sense, but I think that you might be violating the statistical assumptions of the linear models, particularly in the case of the milk proteins. If you only have four possible milk proteins, there is not much data available to use as a variable in a linear mixed model, and you will have the same problem in the bivariate plots (e.g. figure 3). By normalizing against the total number of proteins in a category that is presumably much higher (i.e. the total number of orangutan proteins), you are displaying variation along the x-axis that doesn't really exist. Suppose that for one sample you found 50 orangutan proteins and in another you found 400. However, if you only found 2 or 3 milk proteins the normalized values (e.g. 2/50 vs 2/400) are poorly normalized. This would be less of a concern if you had a higher number of proteins (e.g. for the probiotic bacterial category with ~20 proteins). I understand that of course you need to normalize these data, but this doesn't seem to be an appropriate way of doing so. That said, while I don't think this affects your main question of the timing of weaning, because the presence/absence data is clear, it is more of a concern for the other analyses.

-Also, Student's t-tests might not be valid here. You should use a non-parametric test given the low and uneven number of samples and proteins.

- Are your statistical (LMMs, T-tests) FDR corrected where appropriate?

General:

-I am confused why you are only finding 4 milk proteins. How many are there in total in the database? Also, by using data dependent analysis, you are likely to have a high amount of protein dropout for low intensity proteins. Obviously, I am not asking you to redo things, but would data independent analysis get around some of these limitations?

- How are you categorizing proteins that could have multiple functions? I am thinking particularly of the biological defense proteins.

-Throughout the paper, you use the term "quantitative" proteomics. I don't think that counting the number of proteins like this is really quantitative proteomics. Label free quantification (LFQ) methods rely on spectral counting or calculating protein intensities and this how proteomics readers will expect the analysis to have been conducted. In the supplemental you use the term "approximate quantitative measure", which I think is much more appropriate and should be used throughout the paper.

-I think it would help to include a more direct comparison with the isotopic data you already have. This would be interesting and could make your results more robust.

-Figure 2 is confusing. Please explain in greater detail in the caption. Are there 62 milk proteins shared by A&A and Juveniles and one found in just milk and A/A? Or am I misunderstanding? That seems like an important point.

-Please include the R2, slopes, and p-values (where appropriate) on all bivariate figures

-Why does the x-axis of figure S9 go up to 800 when the data only reach ~200? This gives a distorted slope

Reproducibility:

-When was the database downloaded? Which version of SwissProt did you use?

-Please include the parameters files used for pFind and MaxQuant. Otherwise, it is not possible to confidently reproduce your analyses.

-The Zenodo files are restricted, so I am unable to assess the details of your computational analysis

Version 1:

Reviewer comments:

Reviewer #1

(Remarks to the Author)
The paper is much improved. It is an important contribution to orangutan biology and the study of lactation. There are 3 issues that need to be addressed, relatively minor and easily settled.

Line 42: "…accurately quantify…" overstates your results. Suggest replacing with "…predict…"

Line 89: To me "quantitatively" when used with detecting breastfeeding would imply the amount of milk ingested or at least the proportion of nutrients from milk. I don't see that your study can do that. It can provide better evidence for breastfeeding and I would support that it is fair to say the fecal proteomics method is more sensitive to the occurrence of breastfeeding than other techniques. So better sensitivity, less likely to make a false negative finding, than other techniques used previously. I guess you can defend using "more accurate" but NOT "quantitatively". I really think it is an improvement in sensitivity and decreasing the chance of a false negative result.

Line 232: Replace the word "constant" with "consistent". Your data shows that milk intake consistently occurs over the first 6.5 years but cannot make any claim about the actual amount of milk ingested. It could vary with season and age. Constant implies a set amount of milk ingested over all ages.

Reviewer #2

(Remarks to the Author)
This MS contributes to solving the discrepancy between behavioral observations and previously used lab-based methods using fecal samples to assess milk consumption throughout the many years of offspring dependence in wild orangutans. The authors have managed to revise their MS to address most comments and questions of raised by the reviewers. Apart from a few editorial suggestions for minor changes, I warmly recommend publication of this MS.

Detailed suggestions
Line 38 to be on the safe side: change to '...lowest reported infant mortality rate...'

Line 41 add 'a': we used a novel method

Line 42 Since you could show the presence of milk intake but did not measure quantities replace 'quantify' by 'document'

Line 44 immature instead of juvenile (see ongoing discussion on comment R37)
R37 and lines 106-7 The use of the terms 'juveniles' for pre-weaned immatures and 'adolescents' for all weaned immatures will be confusing for many colleagues and most readers unfamiliar with orangutans. The authors could of course still use their own terminology for age classes as long as specified in the methods with the relevant reference in the main text and not only in the supplementary file.

Line 44 AND line 232: instead of continuously, which would mean immatures drink milk non-stop: use consistently or daily or regularly?

Line 48 at least 'the mothers in this study' were not pregnant yet... It is often reported that in the wild mothers conceive before the previous infant has stopped suckling, but you tested this for these individuals.

Line 144 The oldest sample suggests the sample itself was old, whereas it was the individual that was the oldest... Change to: Lactase was detected in the feces of a 7.1 years-old individual (Figure 1), in the absence of milk-specific proteins. Could add a sentence here: Both milk-specific proteins and lactase were present in the second oldest individual of 6.5 years of age.

Figure 1, Question: How do you explain the absence of lactase in some of the samples with milk-specific proteins? Low quality of the sample? not checked? or a true absence?

Line 146: Would it be possible to add behavioral data on suckling for these 2 oldest individuals – until what age was this observed?

Line 179 'and its benefits on reproductive success and offspring survival' No data has been shown on either by this study - leave out this part of the sentence.

Line 185 (see also response R8) long lactation periods are closely related to long interbirth intervals: in fact in ref 9 - Fig 4 it is shown that wild orangutan females (across all study sites) who lost their preweaned infant tended to conceive their next infant within 6 months, irrespective of age of death of the dependent infant, suggesting a strong link between a mother's active lactation status (at least for the first 4 years) and ability to conceive. Obviously, the offspring benefits from prolonged lactation, especially where food conditions irregularly fluctuate, as in forests in Southeast Asia.

Line 234 Since this study does not provide quantitative measurements of milk vs. solid food intake and the protein content of most plant foods will be lower than that in milk, it would be safer to say that milk is not the only source of protein (instead of not he primary source). Milk's relative importance is still high for a 3-year old, but likely to decline with increasing age and increasing reliance on solid food...

Reviewer #3

(Remarks to the Author)
I appreciate the efforts that the authors have taken to address my comments, which were lengthy. I think the manuscript is much improved, and I believe it is an outstanding paper! That said, I still have a few remaining concerns, particularly about statistical issues.

R61: The authors are correct that mixed models do not need to have even sampling of groups to run. What I think is being neglected is the fact that LMM results are more likely to be biased when there is uneven sampling AND very low sample sizes. Just because you can run the model doesn't mean that the data are appropriate for it. I definitely understand the challenge of collecting an ideal sample from wild primates and agree that these are extraordinary data and really interesting results. My point here is simply that you need to add a note of caution in the paper about the limitations of working with small samples and uneven distributions.

R67: "Unfounded" might be a bit strong here. Great news if I am wrong, but I was under the impression that MaxQuant will place matches to multiple isoforms of the same protein on separate rows of the proteinGroups table when the peptide matches to isoform-specific peptides (as opposed to grouping them into a single protein group when there are not).

R74: I think here I might have been imprecise in my wording. I understand your normalization procedure. What I mean by "not a lot of data" was that ultimately you are working with a very small number of milk specific proteins. Regardless of how you normalize them, you are still transforming values (the number of milk-specific proteins) that originate on scale of 1 to 4. Your normalized values have a much larger range that allows you to use a linear model, but that doesn't mean that the data are appropriate for the statistical expectations of the model. My point here is that you have what seems like a wide range of continuous data for your x-axis in figure 3, but that's largely the result of the normalization procedure more so than actual variation that exists in the the number of proteins recovered. If you only had one protein of interest in 100 samples, you could also normalize it to have 100 different "continuous" values depending on what you normalize it against. That wouldn't change the fact that your original data are not appropriate for a linear model. My concern here is that you are violating the conditions of the linear model and drawing a line through data that you shouldn't be drawing a line through. I am NOT suggesting that your results are spurious, but I think you need to be more cautious in how you report the linear model results. I understand the difficulty of getting orangutan samples and don't think that the limitations of you data should get in the way of publishing important data, but I think you should be more cautious in your reporting.

R77: I do not agree. This is confusing and needs to be clarified. The fact that the word "quantitative" is widely used out side proteomics is not a valid justification. This is a proteomics paper that will be read by people both with and without proteomics experience. I don't think it's very much to ask that you include one sentence in the main paper clarifying the fact that you are not doing quantitative proteomics.

Reviewer #4

(Remarks to the Author)
I co-reviewed this manuscript with one of the reviewers who provided the listed reports. This is part of the Communications Biology initiative to facilitate training in peer review and to provide appropriate recognition for Early Career Researchers who co-review manuscripts.

It is apparent that the authors have thoroughly addressed the comments submitted in the review process. We are of the opinion that this paper should be published, as we the use of noninvasive sampling efforts to confirm lactation period is a strong contribution to the field of reproductive biology. The edits in framework makes the paper's contribution more evident,

while the methods and study design have been consistently excellent. However, before it can be published it is important to clarify a point that will make the article abstract more accurate.

In Line 39, the addition of the word possible is beneficial to modifying the phrase “increases maternal interbirth intervals” but is not appropriate for modifying the phrase “promotes offspring health”. There is sufficient data to support that longer lactation promotes offspring health and is a statement you make in the subsequent introduction. The sentence should be modified to say “promotes offspring health and possibly increases maternal interbirth intervals”.

Open Access This Peer Review File is licensed under a Creative Commons Attribution 4.0 International License, which permits use, sharing, adaptation, distribution and reproduction in any medium or format, as long as you give appropriate credit to the original author(s) and the source, provide a link to the Creative Commons license, and indicate if changes were made.
In cases where reviewers are anonymous, credit should be given to 'Anonymous Referee' and the source.
The images or other third party material in this Peer Review File are included in the article’s Creative Commons license, unless indicated otherwise in a credit line to the material. If material is not included in the article’s Creative Commons license and your intended use is not permitted by statutory regulation or exceeds the permitted use, you will need to obtain permission directly from the copyright holder.
To view a copy of this license, visit https://creativecommons.org/licenses/by/4.0/

Responses to Reviewers' comments

We would like to thank all of you for carefully considering our manuscript and for providing valuable and constructive comments. We did our best to address all of them by editing some parts of the main text and the SI files. Additionally, certain sections of the manuscript have been revised to enhance readability, and these changes do not impact the study's argument.

Dear Dr Tsutaya,

We are, again, very sorry about the delay in obtaining referee feedback on your submission. Your manuscript entitled "Continuous and prolonged breastfeeding shapes the early life history of orangutans" has now been seen by 3 referees, whose comments are appended below. I think you will agree that, despite the delay, the referees have provided very thorough and constructive reports that reflect the considerable interest in your study. You will see from their comments below that while they find your work of interest, some important points are raised. We are interested in the possibility of publishing your study in Communications Biology, but would like to consider your response to these concerns in the form of a revised manuscript before we make a final decision on publication.

We hope you will find the referees' comments useful as you decide how to proceed. Should further analysis and substantial text revision allow you to address these criticisms, we would be happy to look at a revised manuscript. In particular:

1) Please revise the conceptual framing according to comments from Reviewer #1 to focus on the potential adaptive significance of extended lactation in orangutans (rather than general life history) and use more precise language to avoid implying selection, when this is not explicitly shown. (per Reviewer #1, "this study provides no data that selection acted to increase the interbirth interval; just that it wasn't selected against.)

Response (R) 1: Thank you for your suggestion. We have deleted the sentence dealing with K-strategy, avoided ambiguous words that unintentionally imply selection, and focused on the specific aspects of orangutans' life history (i.e., long dependence of immature individuals and lower juvenile mortality rates).

Main text, lines 49-50, 66-67, 74-76, 79-81, 179-180, 193-194, 199-200

-----

2) Please focus the introduction more prominently on the unique contribution and value of proteomics in this study, as suggested by Reviewer #2 (more accurate assessment of milk intake in wild primates compared to conventional behavioral proxies); also, consider replacing "breastmilk" with "milk" based on comments from Reviewer #2 that the term is typically used for humans to distinguish it from milk from other sources.

R2: We have added the statements regarding the contribution of fecal proteomics as a novel tool in the title, introduction, and discussion, accordingly. Also, the word "milk" has been used

instead of “breast milk”, and the latter has explicitly been used when the subject is human milk.

Main text, lines 1, 41, 88, 96-100, 178, 238-240

-----

3) If possible, please consider including a more direct comparison with isotopic data as suggested by Reviewer #3 (this could be included in main text or as supplement). Please also address the comments of Reviewer #3 regarding uneven sampling by age/sex and the suitability of the normalization procedure for data used in linear models, including performing any additional analysis as necessary.

R3: Direct comparison between the stable isotope ratios and milk-specific protein identification for individual fecal samples has been added as Supplementary Figure S9. Since mixed models account for random effects, the uneven sampling among individuals is appropriately addressed in this study. Regarding the normalization procedure, please see R74.

Main text, lines 149-150; SI, Fig. S9

-----

4) Reviewers also asked for more detailed methods: please note that we do not impose word limits and all methods should be described in enough detail so that they may be reproduced. Please also note that, per journal policy, all sequencing data must be deposited to a suitable repository (for a list of suitable repositories, please see: https://www.springernature.com/gp/authors/research-data-policy/repositories-mandates/19540364) and made publicly available prior to acceptance.

R4: The detailed methods are described in Supplementary Information. Although repeating the same detailed methods in the main text seems redundant, we have added further details there.

Main text, lines 104-107, 119-121, 124, 149-150, 254, 279-283

We are committed to providing a fair and constructive peer-review process. Do not hesitate to contact us if you wish to discuss the revision or if there are specific requests from the reviewers that you believe are technically impossible or unlikely to yield a meaningful outcome.

If you decide to submit a revised version, we ask that your manuscript complies with our editorial policies. Specifically:

For all graphs depicting a single point value (e.g., mean) with error bars, you must add individual data points or convert the graph to a boxplot or dot-plot to show data distribution.

It’s mandatory to provide access to the numerical source data for graphs and charts either through a repository or by providing the data in a Supplementary Data file (in excel format).

All blots/gels must be accompanied by size markers in every figure panel. Uncropped and unedited blot/gel images must be included as Supplementary Figure(s) in the Supplementary Information pdf.

Please ensure that you have complied with the data deposition policies at the Nature Portfolio, please see here.

Please ensure that you have complied with our policies on research involving animals and humans, see here

Please follow the ARRIVE guidelines for reporting animal experiments. Please fully complete an ARRIVE checklist including both the essential and recommended set of items (adding information to the manuscript where needed) and upload this with your revised manuscript.

Please also see our revision checklist for guidance on formatting the manuscript and complying with our policies. A comprehensive guide to our formatting requirements for final submissions is also available for your reference here.

Please use the following link to submit your revised manuscript, point-by-point response to the referees' comments (which should be in a separate document to any cover letter) and any completed checklist:

We hope to receive your revised manuscript within 3 months, but appreciate that every situation is unique. Please take as long as necessary to address these concerns in full, including performing any additional analyses. We look forward to receiving your revised manuscript when it is ready and will not enforce any specific deadline. However, please bear in mind that if the revision process takes significantly longer than six months, we may take into account whether anything similar has been accepted for publication at Communications Biology or published elsewhere in the meantime.

Please do not hesitate to contact me if you have any questions or would like to discuss the required revisions further. Thank you for the opportunity to review your work.

Best regards,

Michele Repetto, PhD
Associate Editor
Communications Biology
orcid.org/0000-0001-5583-7287

On behalf of

Madhava Meegaskumbura, PhD
Editorial Board Member
Communications Biology
orcid.org/0000-0002-4599-6724

R5: Thank you for your instruction. The requirements have been met.

Referee expertise:

Referee #1: animal physiology, milk composition, nonhuman primates

Referee #2: orangutan maternal behaviour

Referee #3: molecular anthropology, primate evolution, proteomics, genomics

Reviewers' comments:

Reviewer #1 (Remarks to the Author):

This study uses fecal proteomics to detect milk protein signatures in fecal samples from wild Bornean orangutans. Fecal samples from juvenile, adolescent, and adult orangutans were analyzed and compared to descriptions of milk proteins in orangutan and other species' milk. Milk proteins (and lactase) were found in all feces from juveniles under 7 years of age, but not in any feces of orangutans older than 7 years. The patterns in milk proteins in juvenile feces confirms nutritive suckling to around 6.5 years of age. This result contrasts with results from previous techniques which showed either minimal breastfeeding after 2.7 years of age or possible "cyclical" breastfeeding by older juveniles. The data are convincing and important to publish.

R6: We appreciate your insightful review comments. For the sake of accuracy, we would like to clarify that the detection of lactase differs from what you mentioned. We understand that the phrasing was chosen to prioritize conciseness over strict accuracy.

-----

2. Overall impression of the work:

The study and experimental design in and of itself is worthy of publication. The paper presents convincing evidence that the technique of using fecal proteomics to detect breastfeeding using feces is a viable and valuable tool. In general, the discussion of orang life history and lactation strategy is useful, but there is a marked reliance on the k-selection theory, an outdated life history theory, to explain orangutan parental investment. The paper would be improved if the authors would be more precise in what they are investigating. This isn't about life history in general. It is about the potential adaptive significance of extended lactation in orangs. Lactation period indicates much more than time between offspring and is an incredibly energetically taxing process that in most species far exceeds gestation in energy required. Tracking weaning periods for offspring can tell us about the parental care needed for offspring success as well as how it is changing with orangutan habitat degradation, parental age, etc.

R7: Revised accordingly. Please see R1.

The effort to frame the importance of the results around lactation related infertility or amenorrhea and extended interbirth intervals is overstated. Lactation related amenorrhea is a consequence of lactation in most primates (not the callitrichid primates), but it is a stretch to state that the natural consequence is somehow an adaptive feature that was under selection. It wasn't selected against, but the selective advantage more likely is benefits to the juvenile. The longer interbirth interval is a consequence that was not selected against. This study provides no data that selection acted to increase the interbirth interval; just that it wasn't selected against.

R8: As you pointed out, although studies in humans have shown that the breastfeeding period is the strongest proximate determinant of interbirth interval, this relationship has not yet been investigated in orangutans. In the previous version of the manuscript, we uncritically applied the mechanism of lactational amenorrhea observed in humans to orangutans; therefore, we have revised that point in the revised manuscript. Specifically, we deleted or carefully rephrased any sentences that suggested a relationship between the breastfeeding period and interbirth interval in orangutans, so that it should be clear that this relationship has not yet been empirically examined.

Main text, lines 49-50, 54-55, 79-81, 178-180, 185

The study itself is worthy of publication and presents novel methodologies and techniques for understanding parental care and lactation in orangutans. With some framework revisions this will be an excellent paper.

R9: Thank you so much for your evaluation.

-----

3. Specific comments with recommendations:

There is no need to bring in "k-selection theory" which is an old (somewhat still useful) framework for looking at life history. The first line of the abstract says what is important: "Orangutans have a slow but secure life history strategy." [Please leave out words like "extremely".] Orangs have the longest interbirth intervals among primates, with low infant mortality. They appear to have extended lactation, with breastfeeding of juveniles extending longer than other great apes.

Those are the important factors that this study examines, and, for the extended breastfeeding claim, actually tests. The authors are urged to be precise, and not wander into statements like: Lines 190-191; "Contrary to chimpanzees and gorillas, humans are characterized by a slow and secure life history, similar to orangutans." Many would argue with humans having a "slow" life history relative to chimps and gorillas, given the shorter interbirth intervals of humans. What is true is that human and orang offspring have a longer period of time where they are dependent on adults. For orangs, juveniles are dependent on their mother, receiving breastmilk as an important resource. For humans, the cooperative childcare system allows longer "dependency". These are interesting points to pursue in this paper that do not need to be disguised as "life history". Life history strategy is bigger than that. Be precise in what you are discussing and do not wander into jargon and theory.

R10: Revised accordingly. Please see R1.

The title is broader than the study warrants. A suggested title would be: "Fecal proteomics as a novel technique to verify continuous and prolonged breastfeeding in the orangutan." The focus of the study is really on proof of extended lactation and less on life history theory in general.

R11: The tile has been revised, considering the reviewers' comments. Actually, fecal proteomics was already published as a novel tool in 2021, and we have omitted words like "novel" from the title.

Main text, line 1

The last sentence of the Abstract is excellent (Lines 52-54). The sentence before is a stretch (Lines 49-51). It is fine to bring in some discussion of human lactation/reproductive strategy in the Discussion, but the paper does not "highlight" the uniqueness of humans. Also, the data presented here do not demonstrate that extended breastfeeding "provides" the physiological and molecular basis for the slow life history of orangutans [again, avoid words like "unique"; this paper does not address all known mammals; and there are plenty out there we do not know much about]. Fair to say that extended lactation is a key component of the slow life history of orangutans. But do we know that it is lactational amenorrhea that stops females from cycling or is it amenorrhea related to low body condition? The extended lactation likely contributes to female low body condition, as it probably is a significant cost, but seasonal food availability would also contribute. Again, fair for the authors to hypothesize that lactation (i.e. the hormonal milieu of lactation) contributes to amenorrhea and a long interbirth interval, but they haven't shown that it is the only or even the most important factor.

R12: These stretched sentences and words have been deleted or rephrased accordingly. Please see R1 and R8.

The last sentence of the first paragraph of the Introduction (Lines 98-100) detracts rather than adds to the paper. There is no reason to bring in "K-strategy". Focus on what the paper investigates. Do orangutans have an extended lactation that would nutritionally benefit older juveniles? At what age does nutritive suckling appear to end?

R13: This sentence has been deleted accordingly. Please see also R1.

Main text, line 61

Delete the first sentence of the second paragraph of the Introduction for the same reason. Edit the first sentence of the third paragraph to take out K-selection and the word "extremely". Words like extremely are value judgements and should be used rarely in scientific publications. Better sentence would be "We hypothesize that prolonged periods of breast milk intake, which provide enhanced biological defense agents in juveniles and contributes to the long interbirth interval for adult females, underly the success of the slow life history of orangutans." Lead with what you are studying and what you demonstrate in your results.

R14: These sentences have been deleted or revised, accordingly. The reviewer's suggestion

has been adopted for the first sentence of the third paragraph. Please see also R1.

Main text, lines 62, 74

The last paragraph of the Introduction is excellent. This is what your paper is showing.

R15: Thank you for your comment.

The Results are clear and easy to follow. Congratulations to the authors for producing interesting and informative data.

R16: Thank you for your comment.

Specific line by line comments and suggestions:

61-65: States that lactation "determines" the reproductive speed and strategy. Determines should not be used in this context, lactation does not necessarily "determine" anything and the reproductive speed can be affected by a myriad of factors, including birth of altricial young. What "determines" speed is not addressed in this paper so should be reframed. Lactation is certainly connected to a slow reproductive speed but what determines what is uncertain until explicitly tested.

R17: The word "determines" has been revised to "influencing".

Main text, line 60

63: "breastfeeding period and older age at the end of weaning represent the so-called "K strategy," which is characterized by a small number of litters, slow growth, and a long 65 life span (1)." Remove "so-called "K-strategy" and replace with slow life history, use of k-strategy distracts from novel findings.

R18: This sentence has been deleted. Please see also R1.

Main text, lines 62-63

66-67: "An extreme K-strategy may be considered the slow but secure life history of orangutans (5, 6)." Suggest that this sentence simply be deleted. Could be reworked to say "Orangutans have a slow but secure life history characterized by longest interbirth intervals among primates, with low infant mortality, associated with an extended lactation".

R19: This sentence has been revised as follows:

“To investigate the significance of breastfeeding and weaning in life history, orangutans are a critical research subject.”

Please see also R1.

Main text, lines 62-63

78-79: Delete “k-strategic” and utilize slow life history instead.

R20: Whole this sentence has been revised according to the reviewers’ suggestion. Please see R14.

79-80: “include prolonged periods of breast milk intake, an increase in infant biological defense agents, and maternal interbirth intervals” Replace with “include prolonged periods of breast milk intake, an increase in infant biological defense agents, and prolonged maternal interbirth intervals”. Sentence is not grammatically correct.

R21: Whole this sentence has been revised according to the reviewers’ suggestion. Please see R14.

163-164: “Bifidobacterium dominates the intestine of healthy breastfed infants and improves their health (26).” Please specify human infants here.

R22: Done

Main text, line 167

176-177: Remove “k-selection” and “extremely”

R23: Done

Main text, lines 178-180

181-183: “The fact that the mothers of breastfed juveniles were not pregnant with their next sibling indicates that long breastfeeding periods are closely related to long birth intervals (3, 4), even in orangutans.” Many animals get pregnant or begin the gestation process during lactation with phenomena such as delayed implantation and sperm storage. It is also generally unnecessary for the paper to emphasize long interbirth intervals as an ultimate cause as it may be a proximate cause, due to effects from maternal body condition, environmental constraints, etc. It is also difficult to argue that it is an evolved trait.

R24: This sentence has been toned downed so that the readers easily understand the relationship between breastfeeding periods and interbirth intervals is yet to be proved in orangutans.

Main text, lines 184-186

185-186: “which facilitates the health of immature orangutans.” Instead say “which likely facilitates the health of immature orangutans” as this was not tested in this study.

R25: Done

Main text, line 188

189-190: The real point of this paragraph is that human and orangutan juveniles become competent and independent later than chimpanzee and gorilla offspring and rely on adults. Please reword paragraph.

R26: The second sentence of this paragraph has been revised as follows, accordingly:

“Contrary to chimpanzees and gorillas, humans are characterized by a prolonged period of juvenile dependence and lower juvenile mortality.”

Main text, lines 193-194

198-199: “This study emphasized the two contrasting evolutionary foundations for the similar, “convergent” slow life histories that occur in hominid species.” No it does not. Remove this sentence.

R27: Done

Main text, line 191

209: This may just mean that proportion of nutrient needs met from milk is much lower after 2.7 years. The stable isotope data indicates that the majority of nutrition comes from milk before age 2.7 years. Combined with the novel and important findings from this study the implication is that while milk continues to be ingested after age 2.7 years of age, milk no longer contributes the majority of nutrition for juveniles. Rather, the same foods that the mother eats likely is contributing most of the nutrition. Hence, no measurable difference in stable isotope ratios.

R28: Thank you for your insightful interpretation. Such a scenario has been described as a possible interpretation in a new paragraph.

Main text, lines 230-241

214-218: Similarly, the "cyclical" breastfeeding findings could arise from the probable wide variation in the proportion of nutrient ingestion over seasons from milk versus wild foods. If you assume that milk intake will remain roughly equal over the last few years of lactation, then seasonal changes in other food intake would drive the "signal" for both stable isotopes, as discussed above, and for calcium-normalized barium concentrations laid down in tooth enamel. The authors' data combined with the previous findings build a nice picture of breast milk contributing a variable proportion of nutrition after 2.7 years of age, probably driven by seasonal food supply. The findings are consistent with milk providing a relatively constant amount of nutrients for juveniles, with the juveniles obtaining substantial nutrition from the same foods as the mother, but with large seasonal variation.

R29: Such a scenario has also been described. Please see R28.

225-226: "intervals of several months (3), and no other mammals have been reported to exhibit such cyclical breastfeeding patterns (1)." This is not necessarily true, fur seals and sea lions leave their offspring and stop producing milk, returning to lactate after periods of days and even weeks, this can be said for primates but not for all mammals. Please reword and say "…no other primates have yet been reported to exhibit…". There is still much we do not know about many mammals' lactation so there is always the possibility that there are primates who have cyclical breastfeeding patterns. However, the mechanism given above for producing the same results in stable isotope and tooth enamel barium is far more likely. Those methods rely on a large proportion of nutrition coming from milk. When large amounts of nutrients come into the system from other foods the signal for milk intake will be lost in the noise.

R30: This sentence has been deleted, as the actual milk ejection/consumption is not well studied even for most primate species.

Main text, line 225

233-235: "The results of this study suggest this low reproductive rate partly originates from the physiological and molecular traits of prolonged breastfeeding periods of orangutans." Lactation has not been proven, in this paper or others, to be the ultimate mechanism for low reproductive rate, please remove "partly originates". This sentence can be rephrased to say "The results of this study confirm that orangutans have prolonged breastfeeding periods and maternal investment, which likely contribute to both the high juvenile survival and low adult female reproductive rate."

R31: This sentence has been revised accordingly.

Main text, lines 244-247

236: "… interventions that shorten the breastfeeding period in captive or wild orangutans may

increase the population by ceasing lactational amenorrhea earlier and shortening the interbirth interval (3, 4); however, such interventions would be accompanied by long term negative effects by either completely or partially undermining the short-term and lifelong health benefits of breast milk intake (2).” While this may be an important takeaway from the study, it should be framed as a speculation and not as a main takeaway. Also, in Shumaker et al 2008 (https://doi.org/10.1159/000137705) the authors note that in association with the earlier age at menarche and shorter interbirth intervals of captive orangs there is also menopause at about age 41. Something not seen in the wild. Anthropogenic interventions often have unexpected consequences.

R32: It has been made clearer that this statement is based on inference.
Main text, lines 247-250

The end of the paper should emphasize the confirmation of active lactation period using novel techniques first and then the varying implications (including the one listed here). Emphasizing that the data points to longer lactation periods for reproductive success and offspring survival is essential.

R33: These points have been shown first at the beginning of Discussion.
Main text, lines 178-180

Michael L. Power, PhD
Animal Scientist
Center for Species Survival
Smithsonian's National Zoo and Conservation Biology Institute

Reviewer #2 (Remarks to the Author):

This MS reports on an amazing amount of detailed work and shows how proteomics can be applied to non-invasively collected fecal samples from natural population of wild mammals. This method yields detailed milk-intake-specific results and is a huge improvement over other methods recently used to estimate milk intake in great apes in the wild. Yet, this message deserves more prominence in the title and framing of the MS and I hope the feedback is useful to do that.

R34: Thank you for your comment. The contribution of fecal proteomics has been made clearer. Please see R2.

Strong point: Proteomics applied for the first time to detect maternal milk intake by immatures in a natural population of orangutans. Despite the current small sample size this approach convincingly

documents actual milk consumption by the offspring over almost the complete interval of many (here > 6.5) years before a younger sibling is conceived. The results take away any doubts about the frequently reportedly exceptionally long period of offspring dependence and consequently long inter-birth intervals. In contrast, other recent studies on primates to detect milk intake using isotopes could not separate milk from other animal-based sources of isotopes and thus yielded inconclusive results and dubious interpretations (e.g. Badescue et al. 2017; Smith et al. 2017). Thus, this study is an important contribution to refute such assumptions about 'comfort suckling' or 'cyclical lactation' and the results fully concur with extensive observational data on numerous wild populations.

R35: Thank you for your evaluation.

Weaker point: Even though the abstract is clear, it is not immediately obvious from the title and framing of the introduction that and how proteomic analyses are a great contribution to our understanding of the multi-year lactation pattern of great apes as well as other mammals with slow life history. The slow life history of orangutans is prominent in the title and introduction but was already sufficiently documented in the literature (as also cited by the authors). There is no need to discuss K-strategies etc. mentioning slow life history should suffice to direct the attention to the lactation pattern. However, in my view the MS should immediately draw attention to the advantage of a better documentation of the long and consistent maternal investment through lactation. In the discussion this aspect of the results could also be addressed more clearly and be discussed in relation to the sustained maternal effort (based on time budgets) over most of the long lactation and interbirth period as reported by van Noordwijk et al. 2013 DOI 10.1007/s00265-013-1504-y; and van Noordwijk, Kuzawa & van Schaik 2013 DOI: 10.1002/evan.21368.

R36: Please see R2 for the emphasis on the contribution of fecal proteomics. Please see R1 for the refinement of the theoretical framework employed. Regarding the maternal investment through lactation, the energetic costs of milk production or breastfeeding have been newly added as one of the possible factors affecting the interbirth intervals of orangutans. Thank you so much for introducing us an essential aspect of orangutans' life history.

Main text, lines 58-61, 198-201

General comments

Terminology: infants, juveniles and adolescents

In animal studies the term 'juvenile' is generally used for immatures, which are expected to survive the death of their mother/caretaker but are not yet sexually mature (e.g. Pereira 1993 In Fairbanks & Pereira 1993*). Despite growing evidence offspring survival is not exclusively dependent on weaning stage, it is common practice to use 'infant' for pre-weaned offspring (still receiving milk) and 'juvenile' for fully weaned immatures. Alternatively, the terms dependent vs. independent (or weaned) immatures have been used esp. for orangutans. The term adolescent is used for the developmental stage with rapid hormonal changes into sexual maturity. Without hormonal analyses the onset of this

tends to be hard to recognize in wild primates but is usually considered to start multiple years after weaning in wild orangutans. The age classification used in this MS seem to be based on the faster growth of orangutans in captivity/human-provisioned situations. However, since actual ages of the immatures are known, there is no need to use any of these terms and instead use ages and conclude based on the results whether which individual was still ingesting milk and thus pre-weaned and which individuals were fully weaned. For weaned individuals the only relevant further classification would be whether they have already reached adult body size.

- Pereira, M.E. and Fairbanks, L.A. eds., 1993/2002. Juvenile primates: life history, development and behavior, with a new foreword. University of Chicago Press.

R37: The reviewer's point is reasonable. In situations where data are incomplete, terminology on developmental stages inevitably involves a degree of uncertainty (and in field studies, it is almost impossible to obtain perfect life history data for all individuals). For this reason, we categorized and defined age classes according to estimated age since birth (or, more precisely, first observed date) by following Kuze et al. (2005: AJP 65:353–376). This definition was adopted for the sake of convenience to keep the manuscript concise and straightforward, and it carries no functional implications regarding developmental stages. Therefore, we retained the original terms for age classes. However, we have now explicitly stated that the definition is purely for convenience and does not imply any functional meanings of life history. Some sentences have been revised accordingly.

Main text, lines 105-107

SI, second paragraph of Sample and data collection, Supplementary Materials and Methods

-----

Breast milk: this term is typically used for humans to distinguish it from milk from other sources (animals, plants or industrial infant formulas). For wild mammals, milk is always maternal milk (in rare cases from allomothers) so can just be referred to as milk.

R38: Revised. Please see also R2.

Main text, lines 40, 45, 46, 82, 115, 121, 129, 131, 135, 137, 142, 146, 148, 150, 159, 170, 187, 206, 209, 215, 218, 250

Other comments

Line 69 Confusing sentence: currently orangutans only occur in Sumatra and Borneo however the 3 spp. are separated in distribution 2 in Sum 1 in Borneo. Maybe mention the 3 spp. in previous sentence for easier flow?

R39: Revised, accordingly.

Main text, line 65

Line 73-77 these details seem not relevant for this MS

R40: These details have been retained, as they may not be immediately evident to readers who are not specialists in mammalian life history or orangutans.

Main text, lines 70-72

Line 72 change to suckling from the mother's nipple

R41: Done

Main text, line 73

Line 77 most human populations

R42: Done

Main text, line 74

Line 81-82 This is the main topic of this paper! And deserves more prominence….

R43: This point has been emphasized throughout the manuscript. Please see R2.

Line 83-4 comfort suckling is not observed but assumed… if one cannot easily see whether milk is transferred comfort suckling is an interpretation based on lack of evidence of actual transfer…. In fact, the current study could show whether 'comfort suckling' happens over an extended period in this orangutan population by combining observations suckling with evidence for milk-consumption. (In fact, this study shows that comfort suckling is certainly not happening over a long period…)

R44: The sentence has been revised to make clear it is not observed but assumed.

Main text, lines 78-80

Line 86-9 fully agree! See also Badescu et al. 2025 DOI: 10.1002/ajp.23552 for isotope results based on hair suggesting longer milk intake than previously concluded based on fecal isotopes.

R45: Thank you for providing additional reference. However, since this study focuses on orangutans and does not address chimpanzees, and because it remains uncertain whether the phenomena identified in orangutans can be directly applied to chimpanzees, we decided not to discuss Badescu et al. (2025). It may be worth noting that the last author is currently conducting

a research project using fecal proteomics to more accurately investigate the weaning age in wild chimpanzees.

Main text, lines 82-85

Line 91 Pongo pygmaeus morio: add subspecies. This is relevant since the morio subspecies was long thought to have shorter interbirth intervals than the other subspecies or Sumatran orangutans (even though based on small sample sizes van Noordwijk et al. 2018: reference 6) did not find a consistent difference).

R46: Done

Main text, line 87

Line 118 Figure 1 shows variation in the presence of actual milk-specific proteins for all dependent immatures without a clear age effect. This seems consistent with a rather steady milk intake over many years in line with the sustained maternal effort (fluctuating with fruit abundance but not offspring age) over most of the long lactation period reported by van Noordwijk et al. 2013a&b. (see doi above). Even though their study was on a different population of orangutans, it would be appropriate to discuss these results in relation to their results on maternal effort (feeding time) over the course of the long interbirth intervals of orangutans.

R47: Such an aspect of orangutans' life history has been added accordingly. Please see R36.

Main text, lines 58-61, 198-201

Line 179 please mention that ref 20 is based on the same population which makes the consistency as strong as possible. Of course it is also consistent with other populations….

R48: Done

Main text, line 124

Line 180 suggests that Danum Valley may be exceptional even among orangutans, which it is not (in this respect): for all populations included in van Noordwijk et al. 2018 contributors also mention suckling until shortly before or even after the mother conceived a subsequent offspring. With an overall average of 7.6 years between births, many orangutans suckle for more than 7 years. It's not clear how relevant human reference 3&4 are here.

R49: We do not intend for Danum Valley to be exceptional. The sentence has been revised to make this point clear.

Main text, line 183

Line 190 Explain what you mean by secure life history? Low infant mortality?

R50: This sentence has been revised as explained in R26, and the word “secure” has been replaced. The word “secure” in Abstract has been deleted.

Main text, lines 37, 193-194

Line 198-199 What is meant by ‘two contrasting evolutionary foundations for the similar, “convergent” slow life histories that occur in hominid species.’ All great apes have the same foundation of multi-year lactation with a long period of overlap between increasing ingestion of solid food with a consistent buffer of maternal milk. Yet humans managed to reduce the period of offspring milk-dependence despite their increased requirements due to larger brain size) thanks to active food processing (cutting, mashing, cooking etc.) as well as active provisioning and alloparents. (e.g. van Noordwijk Kuzawa van Schaik 2013; van Schaik 2016 The Primate Origins of Human Nature, Wiley& Sons).

R51: This sentence has been deleted by adopting the comment by Reviewer 1. Please see R27.

Main text, line 201

Line 200-230 Yes! proteomics is the more reliable tool to assess milk-intake. Previous studies based on isotopes in fecal matter and teeth have lead to confusing and premature conclusions, probably because these studies did not take actual food intake and age-dependent digestibility of solid food into account. Your results should restore trust in long term behavioral observations even when some lab-based analyses lead to unlikely conclusions. Thank you!

R52: Thank you for your evaluation.

Line 236-240 Such manipulation of the natural reproductive pattern is known for captivity and ‘rehabilitation’ projects but should have no place in conservation efforts of a species in its natural habitat and does not respect orangutans’ complex social organization. The suggestions might increase the number of orangutans alive (but see negative consequences such as higher infant mortality under the proposed circumstances reported by Kuze et al. 2012 (DOI 10.1007/s10329-011-0285-z) and Galdikas & Ashbury 2012 (DOI 10.1007/s10329-012-0331-5). However, such manipulation would not increase any natural and healthy population! Currently, the main problem for orangutan conservation is a lack of large stretches of safe habitat where orangutans can maintain their own lifestyle. The main message for conservation from this paper is that existing populations need protection because their slow life history implies they can hardly recover from any population crashes…

R53: We never intend to recommend such anthropogenic intervention, as the reviewer may be aware. This sentence considers a hypothetical situation. Therefore, the sentences have been revised to avoid misinterpretation.

Main text, lines 244-250

References

I respect that references often reflect a personal history and preference of the authors, nevertheless in many cases alternative references may be more appropriate e.g.

Ref 1: See Lee 1996 in Evol Anthro: https://doi.org/10.1002/(SICI)1520-6505(1996)5:3<87::AID-EVAN4>3.0.CO;2-T

Ref 4 Bongaarts' parameters seem chosen to compare human populations – it would be more relevant to refer to primate literature here. Specifically, orangutan literature with insights into their social organization which is very different from human conditions. At least the need for an extended period of mating to allow for meeting and mating with many different males is relevant here e.g. Knott et al. 2010 doi: 10.1098/rspb.2009.1552 ; Kunz et al. 2021 https://doi.org/10.1016/j.anbehav.2021.09.012 ; Kunz et al. 2023DOI: 10.1002/ajp.23535 ; Scott et al. 2019 https://doi.org/10.1007/s10764-019-00097-8 ; van Noordwijk et al. 2023 https://doi.org/10.1007/s00265-023-03407-6

Ref 16 Miller et al . on human seems not the most relevant reference to make the point about unreliable observation for primates due to missing overnight or high in canopy suckling… see e.g. Badescu et al. 2017; van Noordwijk et al. 2013

R54 Thank you for indicating valuable references. Lee (1996) is an essential publication in animal life history. Regarding the determinants of interbirth intervals, the suggested publications address reproductive conflicts and strategies between adult females and males, and are therefore considered out of scope for this study. Miller et al. (2013) have been omitted.

Main text, References

Figures

Fig 1 vs. Fig S1 explain the difference between normalized intensity and normalized counts?

R55: Done. Please note that the definition was provided in the first paragraph of Statistical tests, Supplementary Materials and Methods.

Main text, line 438-439

Fig 2 please label the y-axis

R56: Done

Main text, Figure 2

Fig 3B: x-axis label needs fixing at right end ‘Adult’

R57: Done

Main text, Figure 3

Fig S3-6 please provide legend explaining the graphs including the meaning of the colors

R58: These are tandem mass spectra and the corresponding peptide sequence. They are rather raw data of mass spectrometry output and are typically used without specific explanations in the proteomics community. Considering a broader readership, however, brief explanations have been added to each figure.

SI, Fig S3-S6

Fig S8 label y-axes as probiotic bacterial proteins

R59: Added, together with other similar figures.

SI, Fig S2, S7, S8, S10

Reviewer #3 (Remarks to the Author):

The authors use fecal proteomics on a wild population of orangutans to identify the timing of weaning across 10 individuals of various ages. They also identify the number of dietary and potentially probiotic bacterial proteins, which they associate with the presence of milk in the juvenile diet. I think this is an outstanding paper that uses a novel technique to address an interesting important ecological question. It is also an advancement over previous fecal proteomics research in that it extends the method to a free-ranging population opening up many possibilities for molecular ecological research. That said I have many questions about the data analysis that should be addressed and clarified in the paper where appropriate.

R60: Thank you for your comment.

Sampling:

-There is uneven sampling among the age and sex categories. For instance, all non-juvenile samples are female. Can you justify why this does not affect your results? If you run the same analyses with only female juveniles, do you get comparable results? Also, you have a variable number of samples per individual. While this shouldn't affect the broader result of milk proteins being absent in adolescents and adults, it could affect your linear models. It would also helk the reader to include a supplemental table with the number of samples per individual.

R61: Evenly distributed ideal sampling is virtually impossible in solitary wild orangutans, whose encounter rates and sampling efficiency are greatly lower than those of African great apes. Nevertheless, we would like to clarify that mixed models consider random effects, and thus, uneven sampling among individuals does not affect the results of the analysis. A supplemental table with the number of samples per individual has been newly created as Supplementary Table S12.

SI, Table S12

Proteomics database searches:

-It is difficult for the reader to assess what is in the protein databases. SwissProt is a large database, but the reader doesn't really know what you are searching against. For example, how many plants bacteria, etc? Please provide a supplemental table that describes the number of major taxa.

R62: Swiss-Prot is commonly used in the proteomic community, and the rough content has been shown as a reference.

SI, first paragraph of Data analysis, Supplementary Materials and Methods

-----

-Please also provide a supplemental table that lists the number of proteins in the databases that you are assigning to each category you each of your investigated categories. For example, how many milk proteins are there in the database (not just the number you found)? How many biological defense proteins, etc?

R63: Any sequence present in the database will yield a hit and appear in the results; thus, the fact that a protein was identified is sufficient. Conversely, proteins absent from the database will not produce hits and therefore will not appear in the results. In principle, one would have to list those absent from the database, but such an endeavor would constitute an impractical attempt to prove absence, nevertheless requiring an overwhelming amount of effort. All great apes share the same set of milk-specific proteins (i.e., three caseins and lactalbumin with species-specific sequence variations), and since the orangutan sequences of these proteins are included in the database, it follows logically that the corresponding orangutan sequences should be detected regardless of how many other milk proteins are present in the database. The same reasoning applies to the biological defense proteins of orangutans. Although we cannot determine

precisely which bacterial or plant proteins are represented in the samples, Swiss-Prot is sufficiently comprehensive, and because protein sequences display higher interspecific similarity than DNA sequences, we are confident that our analysis identified the proteins present in the samples in a realistically comprehensive and reproducible manner. See also R62.

-Why is SwisProt the appropriate choice for the database? Other metaproteomic studies have opted for broader initial databases (the entire UniProt, NCBI, databases) or more targeted choices (selecting only the microbes found in microbiome studies of the same species).

R64: We chose to use Swiss-Prot because we aimed for a conservative yet reliable identification based on high-quality protein sequences. Since we did not perform metagenomic analysis, a more targeted database was not available.

-Why do you use pFind for the first database search and MaxQuant for the second? Many different database search tools are used in proteomics and metaproteomics. It is not clear to me why MaxQuant should be considered more accurate (or appropriate for the second search) than pFind or other programs.

R65: By sequentially applying two software programs, we aimed to enhance the robustness and efficiency of protein identification. This point has been newly explained.

SI, second paragraph of Data analysis, Supplementary Materials and Methods

-----

-Why do you set the threshold for 2+ razor-unique peptides in the second database search. If a single peptide is identified with high confidence, isn't the probability of if not being correct is extraordinarily low, even in the context of a large database? This seems like an unnecessarily strict threshold.

R66: Threshold of more than two razor+unique peptides is commonly adopted in mass spectrometry proteomics. We aimed at conservative yet reliable protein identification in this study. Therefore, more stringent criterion was adopted. This point has been clarified.

SI, third paragraph of Data analysis, Supplementary Materials and Methods

-----

-How do you address the presence of multiple isoforms in your database? Proteomes often include multiple isoforms of the same protein which can bias your searches.

R67: Since razor+unique peptide count in the output of MaxQuant deals with the inflation of protein identification due to the peptide sequences shared by multiple proteins, including isoforms, the reviewer’s concern is unfounded.

-Why did you include proteomes from multiple species of orangutans when you only have one species in your database? I would think it makes sense to just use your target species.

R68: Because the number of registered proteins for Bornean orangutans is limited and does not cover the entire proteome, we also used the entire proteome of Sumatran orangutans in combination.

-What is the source of the contaminants database?

R69: The sources have been shown. They are built into the respective software.
SI, first and second paragraphs of Data analysis, Supplementary Materials and Methods

-----

-Please clarify why you use an FDR of 1% for the peptides and 100% for the proteins in the initial database?

R70: The reason for applying different filtering criteria is that, while peptide identification was performed with high accuracy, for proteins, we did not impose an FDR cutoff; instead, we wished to include all potentially identifiable proteins in the database for the second search.
SI, first paragraph of Data analysis, Supplementary Materials and Methods

-----

-Please justify your choice of fixed and variable modifications. Are these choices made based on the type of sample, extraction method or something else?

R71: The fixed PTM was determined from the experimental procedures. The variable PTMs can frequently be observed in degraded proteomes like archaeological remains and feces. Also, they represent the PTMs most frequently identified in the open pFind search, as well as a PTM essential for identifying collagens expected to be abundantly present in the fecal samples. Such an explanation has been added.
SI, second paragraph of Data analysis, Supplementary Materials and Methods

-----

Statistics

- The underlying assumption about your normalization approach as described in the supplement makes sense, but I think that you might be violating the statistical assumptions of the linear models, particularly in the case of the milk proteins. If you only have four possible milk proteins, there is not much data available to use as a variable in a linear mixed model, and you will have the same problem

in the bivariate plots (e.g. figure 3). By normalizing against the total number of proteins in a category that is presumably much higher (i.e. the total number of orangutan proteins), you are displaying variation along the x-axis that doesn't really exist. Suppose that for one sample you found 50 orangutan proteins and in another you found 400. However, if you only found 2 or 3 milk proteins the normalized values (e.g. 2/50 vs 2/400) are poorly normalized. This would be less of a concern if you had a higher number of proteins (e.g. for the probiotic bacterial category with ~20 proteins). I understand that of course you need to normalize these data, but this doesn't seem to be an appropriate way of doing so. That said, while I don't think this affects your main question of the timing of weaning, because the presence/absence data is clear, it is more of a concern for the other analyses.

R74: There may be a misunderstanding in the reviewer's comments, so let us first clarify the basics. The explanatory and response variables in the LMM are continuous, normalized counts of proteins in each category. Therefore, whether there are only four or as many as fifty proteins in a given category, both are treated as a single variable in the LMM. Thus, the concern raised by the reviewer—"there is not much data available to use as a variable"—is unfounded.

Furthermore, while the reviewer raises an issue with normalization, there is nothing problematic about this approach itself. Normalization is performed to cancel out random variation and enable highly accurate detection of biologically meaningful variation. The number of proteins constituting the proteome of a specific tissue does not vary greatly (e.g., housekeeping genes/proteins). On the other hand, at the proteome level, the total number of proteins identified varies with experimental factors such as extraction efficiency in the lab and ionization efficiency in the mass spectrometer (see Fig. S10). This random variation equally affects both the proteins of interest and all other proteins. In contrast, the biological phenomena for which the proteins of interest serve as proxies affect only those proteins. Thus, by dividing the absolute number of proteins detected in the category of interest by the total number of proteins identified (i.e., normalization), one can specifically examine biologically meaningful variation. Following the example provided by the reviewer: if, in a sample where 400 orangutan proteins were detected, two milk-specific proteins were found, then in a sample with fifty orangutan proteins identified, the expected value for the number of detected milk-specific proteins would not be two, but 0.25. With normalization, results are directly comparable on a standard scale (i.e., 0.25/50 = 2/400 = 0.01). It should also be noted that, in practice, the number of orangutan proteins identified in this study does not exceed 250 (Fig. S10), making the reviewer's scenario only partially realistic.

More fundamentally, the real issue in this comment is the extent to which the probabilistic false negatives inherent to protein identification may influence results when the number of proteins in the category of interest is small. In proteomics, proteins in a sample may not be detected (i.e., false negatives) due to, for example, poor extraction efficiency in the lab and poor ionization efficiency in the mass spectrometer. Assuming that this occurs randomly, its practical importance depends on the number of target proteins. If only one protein is targeted, and it happens to be a false negative, the false negative affects 1/1 of the protein, and the impact on overall results is 100%. In contrast, with a larger target number—say, 20 proteins—a single random false negative affects only 1/20 of the proteins (5%) and the overall impact is minimal.

Taking lactase as an example, which is presumed to be expressed in all juveniles below a certain age, 2 of the 20 juvenile individuals did not yield lactase detections (Figure 1); the false negative rate is thus estimated at approximately 10%. For the milk-specific protein category mentioned by the reviewer, which includes only four proteins, a single false negative would affect 1/4 of the proteins (25%). If the 10% false negative rate for lactase is applied, the probability that all four proteins in this category are false negatives is $0.1^4 = 0.1\%$. Therefore, if a binary standard (i.e., whether milk-specific proteins are detected) were used, one could accurately estimate breastfeeding/weaning status in 999 out of 1000 samples. On the other hand, an unknown degree of variance due to random false negatives is introduced into normalized continuous variables. The width of the predicted intervals shown in Figure 3 likely reflects, in part, such effects. Nevertheless, even taking this into account, this study found significant associations between the normalized counts of milk-specific proteins and those of biological defense proteins and probiotic bacterial proteins. This strengthens rather than weakens the study's argument.

Some of these points have already been discussed in the Supplementary Text S2. Part of the discussion above has been added to Text S2. Thank you so much for raising an important issue.

SI, third paragraph of Supplementary Text S2

-Also, Student's t-tests might not be valid here. You should use a non-parametric test given the low and uneven number of samples and proteins.

R73: We did not perform a Student's t-test, and the confusing sentence has been deleted.

SI, fourth paragraph of Data analysis, Supplementary Materials and Methods

-----

- Are your statistical (LMMs, T-tests) FDR corrected where appropriate?

R74: Since there are no multiple independent trials in this study, this does not apply.

General:

-I am confused why you are only finding 4 milk proteins. How many are there in total in the database? Also, by using data dependent analysis, you are likely to have a high amount of protein dropout for low intensity proteins. Obviously, I am not asking you to redo things, but would data independent analysis get around some of these limitations?

R75: We would like to clarify that the "milk-specific proteins" are not equal to "milk proteins". Milk contains hundreds of proteins (Cleland and Power, 2022), but most of them are expressed in other biological tissues. Such proteins are not the exact proxy of milk intake. On the other hand, milk-specific proteins, which are exclusively expressed in milk, serve as a precise proxy. The wording has been revised throughout the manuscript.

DIA would be an interesting approach to increase the sensitivity of milk-specific protein detection in a future study.

Main text, lines 150, 159, 209

- How are you categorizing proteins that could have multiple functions? I am thinking particularly of the biological defense proteins.

R76: Except for cancer and other disease states, we classified proteins involved in immunity and inflammatory responses as biological defense proteins. The Supplementary Data S1 lists which proteins are included in this category, ensuring reproducibility.

SI, fifth paragraph of Data analysis, Supplementary Materials and Methods

-----

-Throughout the paper, you use the term "quantitative" proteomics. I don't think that counting the number of proteins like this is really quantitative proteomics. Label free quantification (LFQ) methods rely on spectral counting or calculating protein intensities and this how proteomics readers will expect the analysis to have been conducted. In the supplemental you use the term "approximate quantitative measure", which I think is much more appropriate and should be used throughout the paper.

R77: Although the term "quantitative" is used in the manuscript, it is not employed in the sense of quantitative proteomics. The term "quantitative" is widely used outside proteomics, and we believe that a careful reading of the manuscript makes it clear that it is used in its general sense rather than as specialized proteomics terminology.

-I think it would help to include a more direct comparison with the isotopic data you already have. This would be interesting and could make your results more robust.

R78: The comparison has been added as a new Supplementary Figure S9.

SI, Figure S9

-Figure 2 is confusing. Please explain in greater detail in the caption. Are there 62 milk proteins shared by A&A and Juveniles and one found in just milk and A/A? Or am I misunderstanding? That seems like an important point.

R79: Done. For the general principles of the upset plot, please see Conway et al. (2017).

Main text, lines 448-451

-Please include the R2, slopes, and p-values (where appropriate) on all bivariate figures

R80: This information has been added to the caption of the new Supplementary Figure S10.
SI, Figure S10

-Why does the x-axis of figure S9 go up to 800 when the data only reach ~200? This gives a distorted slope

R81: This was done to match the scales of the x and y axes. By doing so, the magnitude of the slope can be understood more intuitively.
SI, Figure S10

Reproducibility:

-When was the database downloaded? Which version of SwissProt did you use?

R82: The date of download has been added. Swiss-Prot does not have a specific version.
SI, first paragraph of Data analysis, Supplementary Materials and Methods

-----

-Please include the parameters files used for pFind and MaxQuant. Otherwise, it is not possible to confidently reproduce your analyses.

R83: Parameter files have been uploaded to the PRIDE repository.

-The Zenodo files are restricted, so I am unable to assess the details of your computational analysis

R84: Since this manuscript is under review, access to Zenodo is restricted. The accessible URL for reviewers was provided to editors, and the information should have been delivered to the reviewers. An accessible URL has been newly provided here, and please use this information if the reviewer does not receive information.
Zenodo link:
https://zenodo.org/records/17309234?token=eyJhbGciOiJIUzUxMiIsImlhdCI6MTc2MDA2MzYyMCwiZXhwIjoxNzkxNTkwMzk5fQ.eyJpZCI6IjFlNWI0MDUxLTc4OTktNDFlOC1iYzdmLTA0MDQ1NjBmMDdlMyIsImRhdGEiOnt9LCJyYW5kb20iOiI2ZDA3NTM3ZTllODQ5ODBmODU3YThhOWM5NDgxN2IwMyJ9.bJ9bR6MBypJb6Sie9HRtYNf00r6GRMO39bmA1ETXfUf5YoZ1HPngFKy08rZ6Y9c0s_NdpKZ45l8nVSLW-brdNg

Responses to Reviewers' comments

We would like to thank all of you for carefully considering our manuscript and for providing valuable and constructive comments. We did our best to address all of them by editing some parts of the main text.

Dear Dr Tsutaya,

Thank you for your continued patience during the peer review process. Your revised manuscript entitled "Fecal proteomics verified continuous and prolonged breastfeeding in wild orangutans" has now been seen again by our referees, whose comments are appended below. You will see from their comments below that while they find your manuscript of considerable interest and substantially improved, some important points remain. We are interested in publishing your study in Communications Biology, but would like to consider your response to these concerns in the form of a revised manuscript before we make a final decision on publication.

We therefore invite you to revise and resubmit your manuscript, taking into account the points raised. In particular, reviewers have suggested several instances where more precise language and text revisions would be beneficial to readers. Reviewer #3 also suggests including some discussion of caveats given the nature of the analyses with small/uneven sampling in the dataset. We note that Reviewer #2 has requested behavioral data (suckling observations for 2 oldest individuals)-- while we would welcome the inclusion of additional behavioural data, if feasible, it is not a requirement.

We are requesting these text revisions to be submitted in the form of a revised manuscript, however, we do not intend to seek further feedback from referees. Revisions will be assessed editorially, which should result in a more expedited processing time. Please provide a point-by-point response to all remaining comments from the reviewers and highlight/track all changes in the manuscript text file.

We are committed to providing a fair and constructive peer-review process. Do not hesitate to contact us if you wish to discuss the revision in more detail or if there are specific requests from the reviewers that you believe are technically impossible or unlikely to yield a meaningful outcome.

At the same time, we ask that you ensure your manuscript complies with our editorial policies. Specifically:

For all graphs depicting a single point value (e.g., mean) with error bars, you must add individual data points or convert the graph to a boxplot or dot-plot to show data distribution.

It's mandatory to provide access to the numerical source data for graphs and charts either through a repository or by providing the data in a Supplementary Data file (in excel format).

All blots/gels must be accompanied by size markers in every figure panel. Uncropped and unedited blot/gel images must be included as Supplementary Figure(s) in the Supplementary Information pdf.

Please ensure that you have complied with the data deposition policies at the Nature Portfolio, please see here.

Please ensure that you have complied with our policies on research involving animals and humans, see here

Please follow the ARRIVE guidelines for reporting animal experiments. Please fully complete an ARRIVE checklist including both the essential and recommended set of items (adding information to the manuscript where needed) and upload this with your revised manuscript.

Please also see our revision checklist for guidance on formatting the manuscript and complying with our policies. A comprehensive guide to our formatting requirements for final submissions is also available for your reference here.

Please use the following link to submit your revised manuscript, point-by-point response to the referees' comments (which should be in a separate document to the cover letter) and any additional files:

When submitting the revised version of your manuscript, please pay close attention to our Digital Image Integrity Guidelines.

We would like to receive your revision within 4 weeks, but appreciate that every situation is unique. We look forward to receiving your revised manuscript when it is ready, and will not enforce a hard deadline on this revision.

Please do not hesitate to contact me if you have any questions or would like to discuss these revisions further. We look forward to seeing the revised manuscript and thank you for the opportunity to review your work.

Best regards,

Michele Repetto, PhD
Associate Editor
Communications Biology
orcid.org/0000-0001-5583-7287

and

Madhava Meegaskumbura, PhD
Editorial Board Member
Communications Biology
orcid.org/0000-0002-4599-6724

Response (R) 1: Thank you for your suggestion. We have addressed all comments provided by the reviewers accordingly. Adopting the suggestion from the editor, behavioral data were not included.

Reviewers' comments:

Reviewer #1 (Remarks to the Author):

The paper is much improved. It is an important contribution to orangutan biology and the study of lactation. There are 3 issues that need to be addressed, relatively minor and easily settled.

Line 42: "…accurately quantify…" overstates your results. Suggest replacing with "…predict…"

R2: Revised, accordingly.
Main text, line 42

Line 89: To me "quantitatively" when used with detecting breastfeeding would imply the amount of milk ingested or at least the proportion of nutrients from milk. I don't see that your study can do that. It can provide better evidence for breastfeeding and I would support that it is fair to say the fecal proteomics method is more sensitive to the occurrence of breastfeeding than other techniques. So better sensitivity, less likely to make a false negative finding, than other techniques used previously. I guess you can defend using "more accurate" but NOT "quantitatively". I really think it is an improvement in sensitivity and decreasing the chance of a false negative result.

R3: The term "quantitative" has been omitted or replaced, accordingly.
Main text, lines 42, 91

Line 232: Replace the word "constant" with "consistent". Your data shows that milk intake consistently occurs over the first 6.5 years but cannot make any claim about the actual amount of milk ingested. It could vary with season and age. Constant implies a set amount of milk ingested over all ages.

R4: The word "consistent" has been used, accordingly.
Main text, line 241

Reviewer #2 (Remarks to the Author):

This MS contributes to solving the discrepancy between behavioral observations and previously used lab-based methods using fecal samples to assess milk consumption throughout the many years of offspring dependence in wild orangutans. The authors have managed to revise their MS to address most comments and questions of raised by the reviewers. Apart from a few editorial suggestions for minor changes, I warmly recommend publication of this MS.

Detailed suggestions

Line 38 to be on the safe side: change to '...lowest reported infant mortality rate...'

R5: Done.

Main text, line 38

Line 41 add ‘a’: we used a novel method

R6: Done.

Main text, line 41

Line 42 Since you could show the presence of milk intake but did not measure quantities replace ‘quantify’ by ‘document’

R7: The word “predict” has been used based on the comment provided by Reviewer 1.

Main text, line 42

Please see also R2.

Line 44 immature instead of juvenile (see ongoing discussion on comment R37)

R37 and lines 106-7 The use of the terms ‘juveniles’ for pre-weaned immatures and ‘adolescents’ for all weaned immatures will be confusing for many colleagues and most readers unfamiliar with orangutans. The authors could of course still use their own terminology for age classes as long as specified in the methods with the relevant reference in the main text and not only in the supplementary file.

R8: Done.

Main text, line 44

Line 44 AND line 232: instead of continuously, which would mean immatures drink milk non-stop: use consistently or daily or regularly?

R7: The word “consistently” has been used based on the comment provided by Reviewer 1.

Main text, line 44

Please see also R4.

Line 48 at least ’the mothers in this study’ were not pregnant yet... It is often reported that in the wild mothers conceive before the previous infant has stopped suckling, but you tested this for these individuals.

R10: Thank you for your comment. Due to the abstract's length limit, we have not been able

to incorporate your comment. We think your point is obvious, and the priority was lower than that of other revisions in the abstract.

Line 144 The oldest sample suggests the sample itself was old, whereas it was the individual that was the oldest... Change to: Lactase was detected in the feces of a 7.1 years-old individual (Figure 1), in the absence of milk-specific proteins. Could add a sentence here: Both milk-specific proteins and lactase were present in the second oldest individual of 6.5 years of age.

R11: Done.
Main text, lines 147-149

Figure 1, Question: How do you explain the absence of lactase in some of the samples with milk-specific proteins? Low quality of the sample? not checked? or a true absence?

R12: This point has already been discussed in Supplementary Information.
SI, Supplementary Text S2

T Line 146: Would it be possible to add behavioral data on suckling for these 2 oldest individuals – until what age was this observed?

R13: Behavioral data have not been included, based on the comment from the editor. Please see R1.

Line 179 ’and its benefits on reproductive success and offspring survival‘ No data has been shown on either by this study - leave out this part of the sentence.

R14: Done.
Main text, line 187

Line 185 (see also response R8) long lactation periods are closely related to long interbirth intervals: in fact in ref 9 - Fig 4 it is shown that wild orangutan females (across all study sites) who lost their preweaned infant tended to conceive their next infant within 6 months, irrespective of age of death of the dependent infant, suggesting a strong link between a mother’s active lactation status (at least for the first 4 years) and ability to conceive. Obviously, the offspring benefits from prolonged lactation, especially where food conditions irregularly fluctuate, as in forests in Southeast Asia.

R15: Thank you for your valuable comment.

Line 234 Since this study does not provide quantitative measurements of milk vs. solid food intake and the protein content of most plant foods will be lower than that in milk, it would be safer to say that milk is not the only source of protein (instead of not he primary source). Milk's relative importance is still high for a 3-year old, but likely to decline with increasing age and increasing reliance on solid food...

R16: Done.

Main text, line 243

Reviewer #3 (Remarks to the Author):

I appreciate the efforts that the authors have taken to address my comments, which were lengthy. I think the manuscript is much improved, and I believe it is an outstanding paper! That said, I still have a few remaining concerns, particularly about statistical issues.

R61: The authors are correct that mixed models do not need to have even sampling of groups to run. What I think is being neglected is the fact that LMM results are more likely to be biased when there is uneven sampling AND very low sample sizes. Just because you can run the model doesn't mean that the data are appropriate for it. I definitely understand the challenge of collecting an ideal sample from wild primates and agree that these are extraordinary data and really interesting results. My point here is simply that you need to add a note of caution in the paper about the limitations of working with small samples and uneven distributions.

R17: Thank you for the clarification. Mentions to the limitations of working with small samples have been added.

Main text, lines 163-164, 177

R67: "Unfounded" might be a bit strong here. Great news if I am wrong, but I was under the impression that MaxQuant will place matches to multiple isoforms of the same protein on separate rows of the proteinGroups table when the peptide matches to isoform-specific peptides (as opposed to grouping them into a single protein group when there are not).

R18: Yes, it is true in MaxQuant. Isoforms have been treated as such.

R74: I think here I might have been imprecise in my wording. I understand your normalization procedure. What I mean by "not a lot of data" was that ultimately you are working with a very small number of milk specific proteins. Regardless of how you normalize them, you are still transforming

values (the number of milk-specific proteins) that originate on scale of 1 to 4. Your normalized values have a much larger range that allows you to use a linear model, but that doesn't mean that the data are appropriate for the statistical expectations of the model. My point here is that you have what seems like a wide range of continuous data for your x-axis in figure 3, but that's largely the result of the normalization procedure more so than actual variation that exists in the the number of proteins recovered. If you only had one protein of interest in 100 samples, you could also normalize it to have 100 different "continuous" values depending on what you normalize it against. That wouldn't change the fact that your original data are not appropriate for a linear model. My concern here is that you are violating the conditions of the linear model and drawing a line through data that you shouldn't be drawing a line through. I am NOT suggesting that your results are spurious, but I think you need to be more cautious in how you report the linear model results. I understand the difficulty of getting orangutan samples and don't think that the limitations of you data should get in the way of publishing important data, but I think you should be more cautious in your reporting.

R19: Thank you for the clarification. Mentions to the potential caveats in statistical analyses have been added. Please see R17.

R77: I do not agree. This is confusing and needs to be clarified. The fact that the word "quantitative" is widely used out side proteomics is not a valid justification. This is a proteomics paper that will be read by people both with and without proteomics experience. I don't think it's very much to ask that you include one sentence in the main paper clarifying the fact that you are not doing quantitative proteomics.

R20: The term “quantitative” has been omitted or replaced, accordingly. Please see R3.

Reviewer #4 (Remarks to the Author):

I co-reviewed this manuscript with one of the reviewers who provided the listed reports. This is part of the Communications Biology initiative to facilitate training in peer review and to provide appropriate recognition for Early Career Researchers who co-review manuscripts.

It is apparent that the authors have thoroughly addressed the comments submitted in the review process. We are of the opinion that this paper should be published, as we the use of noninvasive sampling efforts to confirm lactation period is a strong contribution to the field of reproductive biology. The edits in framework makes the paper’s contribution more evident, while the methods and study design have been consistently excellent. However, before it can be published it is important to clarify a point that will make the article abstract more accurate.

In Line 39, the addition of the word possible is beneficial to modifying the phrase “increases maternal interbirth intervals” but is not appropriate for modifying the phrase “promotes offspring

health”. There is sufficient data to support that longer lactation promotes offspring health and is a statement you make in the subsequent introduction. The sentence should be modified to say “promotes offspring health and possibly increases maternal interbirth intervals”.

R21: We do not fully agree with the comment provided. Based on the comment from Reviewer 2, this part has not been changed. Please see R15.

Responses to Editor's comments

We would like to thank all of you for carefully considering our manuscript and for providing valuable and constructive comments. We did our best to address all of them by editing some parts of the main text and Supplementary Information.

Regarding the following comment provided by the editor:

From an editorial perspective, we would ask that you further address the below comments from the reviewers. Please provide a brief point-by-point rebuttal letter and marked-up version of the main text outlining the changes made.

In response to points raised by Reviewer #3 in the last round of review, we note that only minimal text edits have been made. We ask that you expand upon these points and include additional rationale/logic to support the analysis in the main text.

L 160: "…although the relatively small sample size in this study may affect the statistical results."

L 173: "… although the relatively small sample size in this study may affect the statistical results."

From an editorial perspective, we would ask that you further address the below comments from the reviewers. Please provide a brief point-by-point rebuttal letter and marked-up version of the main text outlining the changes made.

In response to points raised by Reviewer #3 in the last round of review, we note that only minimal text edits have been made. We ask that you expand upon these points and include additional rationale/logic to support the analysis in the main text.

L 160: "…although the relatively small sample size in this study may affect the statistical results."

L 173: "… although the relatively small sample size in this study may affect the statistical results."

I have copied the relevant points from Reviewer #3 again below, for reference:

R61: The authors are correct that mixed models do not need to have even sampling of groups to run. What I think is being neglected is the fact that LMM results are more likely to be biased when there is uneven sampling AND very low sample sizes. Just because you can run the model doesn't mean that the data are appropriate for it. I definitely understand the challenge of collecting an ideal sample from wild primates and agree that these are extraordinary data and really interesting results. My point here is simply that you need to add a note of caution in the paper about the limitations of working with small samples and uneven distributions.

R67: "Unfounded" might be a bit strong here. Great news if I am wrong, but I was under the impression that MaxQuant will place matches to multiple isoforms of the same protein on separate rows of the proteinGroups table when the peptide matches to isoform-specific peptides (as opposed to grouping them into a single protein group when there are not).

R74: I think here I might have been imprecise in my wording. I understand your normalization procedure. What I mean by "not a lot of data" was that ultimately you are working with a very small

number of milk specific proteins. Regardless of how you normalize them, you are still transforming values (the number of milk-specific proteins) that originate on scale of 1 to 4. Your normalized values have a much larger range that allows you to use a linear model, but that doesn't mean that the data are appropriate for the statistical expectations of the model. My point here is that you have what seems like a wide range of continuous data for your x-axis in figure 3, but that's largely the result of the normalization procedure more so than actual variation that exists in the the number of proteins recovered. If you only had one protein of interest in 100 samples, you could also normalize it to have 100 different "continuous" values depending on what you normalize it against. That wouldn't change the fact that your original data are not appropriate for a linear model. My concern here is that you are violating the conditions of the linear model and drawing a line through data that you shouldn't be drawing a line through. I am NOT suggesting that your results are spurious, but I think you need to be more cautious in how you report the linear model results. I understand the difficulty of getting orangutan samples and don't think that the limitations of you data should get in the way of publishing important data, but I think you should be more cautious in your reporting.

We would like to respond as follows:

Response (R) 61: We have added the following sentences in the Result section of the main text:

> Given the small overall sample size and uneven sampling among individuals, these model-based inferences should be interpreted with caution, and we emphasize the qualitative patterns rather than precise effect sizes.

and

> The same limitations of small and uneven sampling described above for the LMMs apply to this result as well.

Also, the following sentences have been added to the Method section of the main text:

> While LMMs are well-suited for handling clustered and unbalanced data, small and uneven sample sizes can reduce the robustness of the model assumptions and increase the uncertainty of parameter estimates. For this reason, the LMM results should be treated with appropriate caution, and the qualitative pattern, rather than precise effect sizes, should be emphasized.

R67: We have added the following sentence to the Method section of the main text:

> In MaxQuant, peptides are assigned to protein groups, and in our workflow, we treated entries corresponding to isoforms of the same gene as separate protein groups if their unique sequences were identified and MaxQuant listed them as separate entries.

R74: We have added the following sentences to the Supplementary Note 2:

Regarding LMMs, the predictor was ultimately derived from counts of a small set of milk-specific proteins (on an original scale of 0–4) that were normalized to allow comparability across samples. Thus, although the normalization yields a quasi-continuous predictor suitable for linear modeling, the underlying discreteness and limited range of the original data further reinforce the need to treat the LMM results with caution. For this reason, we used the LMM as an explanatory tool that formalizes the observed association between milk-specific and biological defense/probiotic bacterial proteins, rather than to obtain precise effect sizes.
